# Mettl7a alleviated bone loss in osteoporosis mice by targeting the O-GlcNAcylation of Bsp via m^6^A methylation

**DOI:** 10.1093/stcltm/szaf024

**Published:** 2025-06-25

**Authors:** Yantong Wang, Yangyang Cao, Zhipeng Fan

**Affiliations:** Laboratory of Molecular Signaling and Stem Cells Therapy, Beijing Key Laboratory of Tooth Regeneration and Function Reconstruction, Capital Medical University School of Stomatology, Beijing 100050, China; Laboratory of Molecular Signaling and Stem Cells Therapy, Beijing Key Laboratory of Tooth Regeneration and Function Reconstruction, Capital Medical University School of Stomatology, Beijing 100050, China; Department of General Dentistry and Integrated Emergency Dental Care, Capital Medical University School of Stomatology, Beijing, China; Laboratory of Molecular Signaling and Stem Cells Therapy, Beijing Key Laboratory of Tooth Regeneration and Function Reconstruction, Capital Medical University School of Stomatology, Beijing 100050, China; Beijing Laboratory of Oral Health, Capital Medical University School, Beijing, 100069, China; Research Unit of Tooth Development and Regeneration, Chinese Academy of Medical Sciences, Beijing, China

**Keywords:** osteoporosis, Mettl7a, O-GlcNAcylation, m^6^A methylation, extracellular matrix (ECM)

## Abstract

Postmenopausal osteoporosis, a prevalent metabolic bone disease, elevates susceptibility to fragility fractures while imposing substantial healthcare costs and public health challenges. The profound interplay between BMSCs and surrounding extracellular matrix (ECM) proteins, which are highly rich in O-GlcNAcylation, play pivotal roles in the process of osteoporosis. M^6^A methylation plays a crucial regulatory role in the development of osteoporosis, while the crosstalk between m^6^A methylation and ECM O-GlcNAcylation remains mechanistically undefined. Here we found Mettl7a overexpression improved the impaired osteogenic capability of OVX-mBMSCs in vitro. Conditional knockout of Mettl7a in the mesenchyme (Prx1-cre;Mettl7a^f/f^) accelerated bone loss of OVX mice. Mechanistically, Mettl7a promoted mBMSCs osteogenic differentiation by targeting the O-GlcNAcylation of Bsp, an ECM protein. Mettl7a regulated the expression and O-GlcNAcylation of Bsp through m^6^A methylation of Oga. We further demonstrated that Mettl7a-AAV treatment alleviated bone loss phenotype in osteoporosis mice via the O-GlcNAcylation of Bsp. Collectively, our findings reveal novel mechanistic intersections between ECM protein O-GlcNAcylation and m^6^A methylation, advancing the understanding of osteoporotic regulation.

## Graphical Abstract

**Graphical Abstract:**
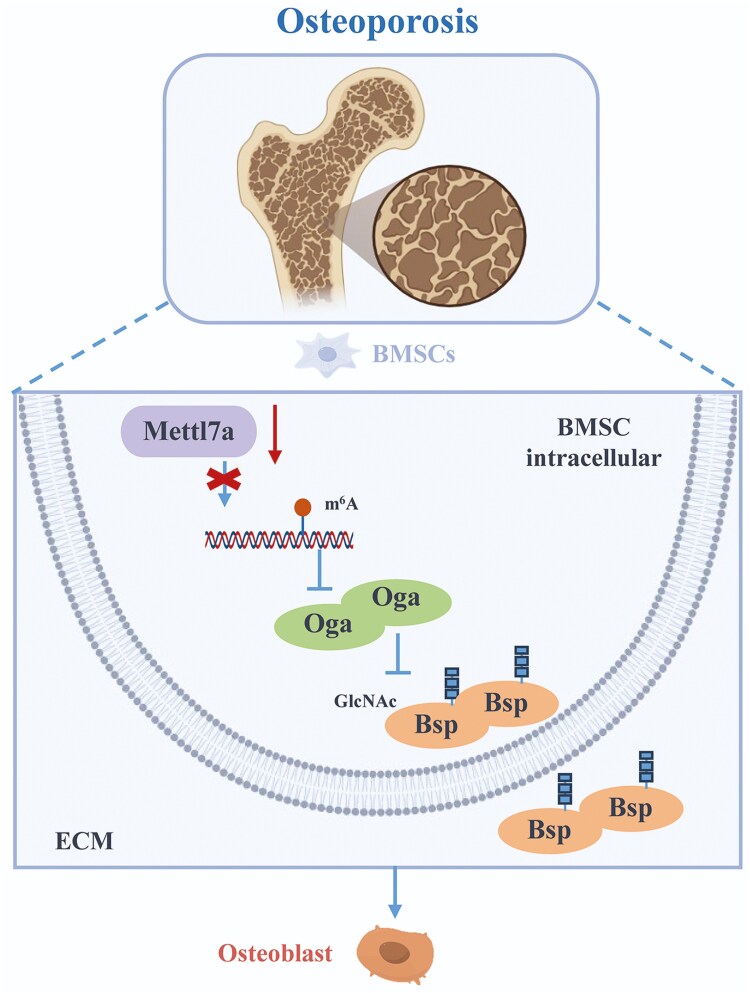

**Significance Statement**

Osteoporosis is one of the most common age-related systemic bone diseases, which seriously impairs the quality of life of older patients and increases the economic burden. Our study identified the key role of Mettl7a in the development of osteoporosis in mice and its regulatory mechanism. Furthermore, Mettl7a gene therapy improved bone loss in the osteoporosis mice. Our study identified the interaction between extracellular matrix proteins O-GlcNAcylation and m6A methylation in bone marrow mesenchymal stem cells. We expect that our study can provide new, effective intervention targets for the treatment of osteoporosis.

## Introduction

As a prevalent skeletal disease, postmenopausal osteoporosis manifests through progressive bone mineral density (BMD) reduction and microstructural degradation, affecting approximately one-third of women population beyond 50 years.^1,2^ Osteoporosis dramatically increases the propensity for fragility fractures, which seriously damages the life quality of elderly patients and increases the socioeconomic burden.^3^ Osteoporosis pathogenesis arises from disrupted bone remodeling homeostasis, particularly the uncoupling between osteoclast-mediated resorption and osteoblast-mediated formation processes.^4^ Current therapeutic strategies for osteoporosis primarily involve osteoclast inhibitors targeting resorption suppression and osteoanabolic compounds promoting bone formation, but prolonged administration carries risks of significant adverse events.^5^ The physiological mechanism of osteoporosis is more than intricate, which involves osteoblast-osteoclast balance, osteoblast-lipoblast balance, and bone immune microenvironment.^6^ Impaired bone marrow-derived mesenchymal stem cells (BMSCs) differentiation towards osteoblasts represents the direct cause of osteoporosis progression. Consequently, systematic exploration of molecular mechanisms governing BMSC functional regulation emerges as the pivotal therapeutic frontier for osteoporosis.^7^

Elucidating the pathophysiological contributions of BMSCs to osteoporotic bone loss is the basis for improving osteoporosis treatment.^8^ Indeed, BMSCs function relies not only on their viability and state themselves but also on the profound interplay between resident or transplanted BMSCs and surrounding niches in osteoporosis microenvironments.^9^ As a dynamic regulatory compartment, the stem cell niche coordinates cellular fate through bidirectional extracellular matrix (ECM)-stem cell crosstalk.^10^ Bone-specific ECM architectures provide stem cells with molecular signaling and biophysical patterning essential for maintaining osteogenic differentiation competence.^11^ In recent years, Emerging evidence positions ECM as a vital regulator governing BMSCs fate decisions.^12,13^ The mitochondrial phosphoenolpyruvate carboxykinase (PCK2), a key gluconeogenic enzyme, was reported to promote osteogenesis in tunable stiffness ECM via glycolysis.^14^ The non-collagen proteins, main components of bone ECM, mainly including bone osteocalcin (OCN), osteopontin (OPN), sialoprotein (BSP), etc.^15,16^ These proteins share a common feature marked by abundant O-linked-β-N-acetylglucosamine (O-GlcNAc) glycosylation (O-GlcNAcylation), suggesting that targeted regulation of glycosylated non-collagen proteins is expected to improve the metabolic balance of bone matrix.^17^

O-GlcNAcylation orchestrates skeletal homeostasis by influencing resorption-formation coupling.^18-20^ As a dynamic post-translational modification (PTM), O-GlcNAcylation involves enzymatic β-N-acetylglucosamine conjugation to proteins at specific serine/threonine residues.^21^ The modification’s dynamic regulation is enzymatically controlled by O-GlcNAc transferase (OGT) -mediated glycosylation and β-N-acetylglucosaminidase (OGA)-driven deglycosylation.^22^ Global intracellular O-GlcNAc modification levels undergo dynamic upregulation during the initiation phase of BMSCs osteogenic differentiation, correlating with the enhanced transcriptional activity of Runt-related transcription factor 2 (Runx2).^23^ The increased O-GlcNAc modification, promoted by the inactivation of OGA and suppressed by the inactivation of OGT, could promote osteoblast differentiation.^24^ Emerging evidence has pointed to the O-GlcNAcylation of ECM as one of the principal modulators of MSCs. Hyaluronan is a linear glycosaminoglycan of ECM synthesized by hyaluronan synthase 2 (HAS2), which mediates MSC proliferation and differentiation. The O-GlcNAcylation stabilized HAS2 in the plasma membrane and increased hyaluronan production.^25^ More importantly, analyses revealed the O-GlcNAcylation-dependent upregulation of non-collagenous ECM components during osteogenic differentiation.^24^ These findings established ECM proteins O-GlcNAcylation as a critical determinant of osteoblast differentiation and a potential therapeutic target in osteoporosis pathogenesis.

O-GlcNAcylation occupies an important position within PTM networks through cross-regulatory relationships with other PTMs.^26^ Recent studies have shown that mRNA methylation may be closely related to the O-GlcNAcylation.^27^ As the most widespread posttranscriptional eukaryotic mRNA modification, N6-methyladenosine (m^6^A) regulates multiple biological processes, including bone development and osteoporosis.^28,29^ The installation of m^6^A modifications is enzymatically catalyzed by methyltransferase complexes, with METTL family members being biochemically characterized as core catalytic subunits.^30^ Emerging evidence proves METTL-family enzymes as critical regulators of BMSCs osteogenic differentiation.^31^ Research found METTL3/14-mediated m^6^A modification governed BMSCs fate decision and osteogenic potentiation.^32-35^ Methyltransferase like 7A (METTL7A) also belongs to the METTL superfamily and has been proven to have methyltransferase activity.^36^

Transcriptomic profiling under metabolic stress identified METTL7A as a critical modulator of hBMSCs viability and osteogenic differentiation.^37^ Current reports have shown that METTL7A reversed bisphosphonates-impaired osteogenic differentiation of orofacial BMSCs.^38^ Besides, studies have predicted a biomarker network for identifying osteoporosis through multi-omics data integration, in which METTL7A is one of the core differential genes.^39^ However, the pathophysiological significance of METTL7A in osteoporosis pathogenesis and its molecular mechanisms require systematic investigation.

This study established Mettl7a as a critical epigenetic modulator in ovariectomy (OVX)-induced osteoporosis, demonstrating its capacity to rescue osteogenic potential in mBMSCs through m^6^A-mediated regulation of Bsp O-GlcNAcylation, thus to alleviated bone loss in osteoporosis mice.

## Materials and methods

### Animals

All experimental protocols involving animals were strictly conducted in compliance with the Beijing Experimental Animal Management Ordinance (Approval of Animal Ethical and Welfare: KQYY-202211-002). All mice involved in the experiment were C57BL/6J mice. Conditional knockout mice of the Mettl7a in Prx1 lineage cells were constructed using the cre/loxp recombination system. The Prx1-cre transgenic line and Mettl7a^f/f^ transgenic line were purchased from Cyagen Biosciences (Suzhou, China). Tissue-specific knockout mice (Prx1-cre;Mettl7a^f/f^) were obtained through intercrossing. Female mice (3 months old) exhibiting normal grooming and walking behavior were randomly selected as observation objects for this study (*n* = 7). The mice osteoporosis model was constructed by bilateral ovariectomy (OVX), as previously described.^7^ Eight weeks after OVX, all mice were euthanized for subsequent analysis. No adverse events such as death occurred during the experiment and observation period. Power analysis was performed to validate the sample size calculations.

### Cell isolation and culture

mBMSCs were obtained from the bone marrow of C57BL/6J mice femur. Briefly, the single-cell suspension was got by repeatedly blowing, and the monocyte was collected by using Percoll (1.082 specific gravity; Invitrogen, Carlsbad, CA), then incubated in standard culture conditions (37°C, 5% CO2) in α-MEM basal medium (Invitrogen) supplemented with 15% heat-inactivated FBS (Invitrogen), 100 U/mL penicillin, and 100 μg/mL streptomycin (Invitrogen). The passage 3-5 cells were used for analysis.

### Viral synthesis and infection

Recombinant lentiviruses encoding Flag-Mettl7a, Mettl7ash, HA-Bsp, HA-Bspmut, and controls were engineered by GenePharma (Shanghai). Viral particles were transduced into BMSCs with 8 μg/mL polybrene, followed by 72h puromycin selection (2 μg/mL). Transduction efficacy was quantified via qRT-PCR. The Mettl7ash sequence was as follows: 5′- GATTCTCCGTGATGTACAATGGGAGCCAACTTCAAGTTCTAT- 3′.

### Reverse transcriptase-polymerase chain reaction (RT-PCR) and real-time reverse transcriptase-polymerase chain reaction (real-time RT-PCR)

Briefly, RNA isolation from mBMSCs was performed using TRIzol-chloroform phase separation, followed by cDNA library construction per established protocols.^40^ Real-time RT-PCR analysis was conducted on Bio-Rad CFX384 and iQ5 systems with QuantiTect SYBR Green Master Mix, employing primer pairs detailed in Supplementary Table S2.

### Western blot

After the cell lysate was quantified by the BCA method, the equivalent protein (30 μg) was separated by 12% SDS-PAGE gel electrophoresis (Mini-PROTEAN Tetra system, Bio-Rad). It was then transferred to 0.45 μm PVDF membrane (Millipore. USA). After the transfer, the PVDF membrane was blocked in 5% skim milk powder at room temperature (RT) for 2 hours and then incubated with primary and secondary antibodies, as previously described.^40^The antibodies included anti-Flag (Cat No. ab207301; Abcam), anti-β-actin (Cat No. ab8226; Abcam), anti-Mettl7a (Cat No. ab79207; Abcam), anti-O-Linked N-Acetylglucosamine antibody [RL2] (Cat No. ab2739; Abcam), anti-Bsp (Cat No. PA5-79424; Invitrogen), anti-HA (Cat No. ab314237; Abcam), anti-histone H3 (Catalog No. ab1791; Abcam). The blots were then acquired by the enhanced chemiluminescence kit (Thermo Fisher Scientific, China).

### Alkaline phosphatase and Alizarin Red detection

For osteogenic induction, cells were seeded in 6 well plates (2 × 10^5^ cells per well), then induced with osteogenesis differentiation medium containing 0.1 μM dexamethasone, 10 mM β-sodium glycerophosphate, 50 μg/mL ascorbic acid. After being induced for 5 days, cells were incubated with 1 × BCIP/NBT buffer (Sigma) for the ALP activity assay. After being induced for 2 weeks, cells were fixed with 4% paraformaldehyde and then stained with 2% Alizarin Red (Beyotime, China). For Ca2 + concentration analysis, the cells stained with alizarin red were destained with 10% cetylpyridinium chloride (CPC) solution at RT for 30 minutes, and the absorbance of the solution at 562 nm was determined.

### Proteome analysis

To further explore the underlying mechanism of Mettl7a on osteoporosis, we analyzed the differentially expressed protein among Sham, OVX + Vector, and OVX + Flag-Mettl7a using label-free quantitative proteomics (Label-free) technology. The quality of the samples was approved by quantification and SDS-PAGE. Qualified samples were used for formal experiments, and unprocessed mass spectrometry data were obtained using a high-resolution mass spectrometer. After building a protein database, the search was performed using analysis software. Subsequently, based on the results retrieved from the database, a quality control analysis was performed at the peptide and protein levels. The proteins with log2 foldchange >1 or <−1 and *P* value <.05 were defined as differentially expressed proteins (DEPs). Finally, differential proteins underwent statistical analyses including Volcano plot, Venn diagram, and KEGG pathway classification.

### Co-immunoprecipitation (Co-IP)

As a previous experimental method,^41^ we performed Co-IP analysis with the Pierce IP Lysis Buffer (Invitrogen) and Pierce™ Direct IP Kit (Thermo Fisher Scientific). The primary antibodies involved were as follows: anti-Mettl7a (Cat No. ab79207; Abcam), anti-O-Linked N-Acetylglucosamine antibody [RL2] (Cat No. ab2739; Abcam), anti-Bsp (Cat No. PA5-79424; Invitrogen), and anti-IgG (Cat No.bs-0297P, Bioss, China).

### Adeno-associated virus construction and injection

The recombinant adeno-associated virus (AAV)-9 encoding Mettl7a (AAV-Mettl7a) was produced by Genomeditech (Shanghai, China). Female C57BL/6J mice were divided into four groups (n = 6/group): sham + vehicle, OVX + vehicle, OVX + AAV-Vector, and OVX + AAV-Mettl7a. At 14 days post-ovariectomy, 2 × 10^12^ viral genomes of AAV-Mettl7a (or AAV-Vector) in 10 μl vehicle (PBS) were intra-tibially injected.^42^ Fluorescence signals were tracked in anesthetized mice using IVIS-100 imaging 14 days post-injection. Tibiae were harvested for analysis 6 weeks after AAV administration.

### Calcein double labeling

Dynamic bone formation was evaluated via sequential calcein green labeling (Sigma, 10 mg/kg body weight), with intraperitoneal injections administered 10 and 3 days prior to euthanasia as previously described.^7^ After euthanasia, the femurs were collected and a 50-μm-thick hard tissue section was performed with a hard sectioning microtome (EXAKT Advanced Technologies, Norderstedt, Germany). Then examined calcein double labeling with a fluorescence microscope and quantified the mineral apposition rate (MAR) with ImageJ software.

### Micro-Computed Tomography (micro-CT) analysis

Femurs and tibias were collected, immersion-fixed for 24 hours, and analyzed using an eXplore Locus SP micro-CT scanner (GE Healthcare, USA) at 70 kV/80 μA, generating 6 μm-thick tomographic slices. Data were analyzed with the data view software (Version 2.1.2), and the quantification parameters were performed by using CTan software (Version 1.15).

### Histology and histomorphometry

Bone specimens (femurs/tibiae) underwent decalcification in 10% EDTA, followed by sequential ethanol dehydration, paraffin embedding, and sectioning at 5 μm. Sections were dewaxed and rehydrated through graded alcohol to distilled water. Hematoxylin-eosin staining and Masson’s staining were performed as previously described.^40^ Microscopy (Zeiss AxioX-4, Zeiss) was used to examine staining after sealing with neutral resin.

### Immunofluorescence analysis

Tissue sections underwent dewaxing followed by PIER-based antigen retrieval using HistoReveal (Abcam, ab103720) for 10 minutes at RT. Permeabilization with 5% Triton X-100 (5 minutes, RT) preceded 0.5% H_2_O_2_ incubation (30 minutes, RT). After 5% BSA blocking (30 minutes, RT), immunostaining was performed with anti-BSP/RL2 primary antibodies and Alexa Fluor-conjugated secondaries (anti-rabbit 488, ab150073; anti-mouse 594, ab150116). Nuclei were counterstained with DAPI (Invitrogen), and fluorescence was visualized using Olympus microscopy.

### Statistical analysis

Data were processed in SPSS 10.0 (IBM Corp.). Two-group comparisons applied unpaired Student’s *t*-tests; multigroup differences were assessed via one-way ANOVA. A significance threshold of *P* < .05 was adopted.

## Results

### Mettl7a overexpression improved the impaired osteogenic capability of OVX-mBMSCs

**Figure 1.**
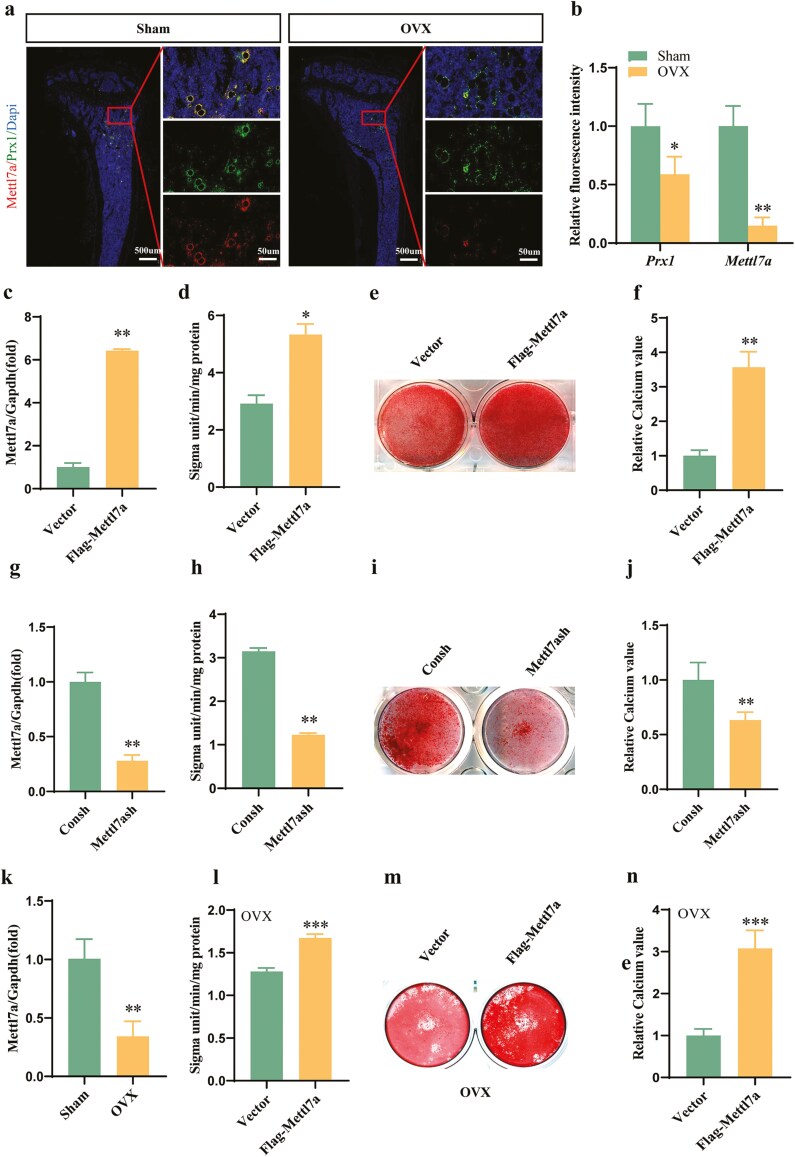
Mettl7a overexpression improved the impaired osteogenic capability of OVX-mBMSCs. (A) Immunofluorescence of Prx1, Mettl7a, and nuclei (Dapi) in the femur of osteoporosis mice induced by OVX. Scale bar = 500 μm(left). Scale bar = 50 μm(right). (B) Quantitative analysis of Prx1 and Mettl7a fluorescence intensity from (A). (C) The overexpression efficiency of Flag-Mettl7a in mBMSCs tested by RT-qPCR. (D) The ALP activity analysis in mBMSCs at induced 5 days. (E) ARS staining of mineralized nodules in mBMSCs at induced 14 days. (F) Quantification of calcium deposition from (E). (G) The inhibition efficiency of Mettl7a in mBMSCs tested by RT-qPCR. (H) The ALP activity analysis in mBMSCs at induced 5 days. (I) ARS staining of mineralization in mBMSCs at induced 14 days. (j) Calcium content quantification from (I). (K) Mettl7a expression in sham and OVX-mBMSCs by Real-time RT-PCR. (L) The ALP activity analysis in OVX-mBMSCs after 5-day osteogenic induction. (M) ARS staining at induced 14 days in OVX-mBMSCs. (N) The relative calcium quality of (M). All data represent mean ± SD. Significance determined by Student’s *t*-test: *P < .05; **P < .01; ***P < .001.

Firstly, in order to explore the effect of Mettl7a on osteoporosis, we determined the Mettl7a expression level in the femur of osteoporosis mice induced by ovariectomy in vivo (Figure 1A and B). Immunofluorescence results showed that Mettl7a was mainly expressed in the bone marrow while barely expressed in cortical bone, and the Mettl7a expression level was significantly downregulated in the OVX group. To investigate Mettl7a expression in MSCs, spatial colocalization analysis was conducted with Paired related homeobox 1 (Prx1), a canonical MSC marker. Immunofluorescence revealed Mettl7a-Prx1 co-expression in bone marrow stromal cells (Figure 1A and B), implying functional roles in MSC lineage determination. Subsequent functional studies evaluated Mettl7a’s regulatory effects on BMSC osteogenic differentiation. Firstly, we overexpressed and knocked down Mettl7a in mBMSCs by lentiviral transfection, respectively (Figure 1C and G). After 5-day osteogenic induction, Mettl7a-overexpressing mBMSCs exhibited elevated ALP activity, contrasting sharply with suppressed activity in knockdown models (Figure 1D and H). Following 14-day differentiation, Alizarin red staining (ARS) and calcium quantitative assays demonstrated increased mineralization capacity in Mettl7a-overexpressing groups (Figure 1E and F, meanwhile knockdown inhibited the mineralization ability (Figure 1I and J). To further delineate Mettl7a’s regulatory effects on BMSCs in osteoporotic microenvironments, primary mBMSCs were isolated from OVX mice and cultured. Quantitative RT-PCR analysis revealed significant downregulation of Mettl7a expression in OVX-mBMSCs (Figure 1K). Next, we overexpressed Mettl7a in OVX-mBMSCs. The ALP activity analysis, ARS and quantitative calcium levels revealed that Mettl7a overexpression in OVX-mBMSCs significantly enhanced osteogenic capacity (Figure 1L-N). All the above results indicated that Mettl7a overexpression improved the impaired osteogenic capability of OVX-mBMSCs.

### Conditional knockout of Mettl7a in mesenchyme cells accelerated bone loss of OVX mice

**Figure 2.**
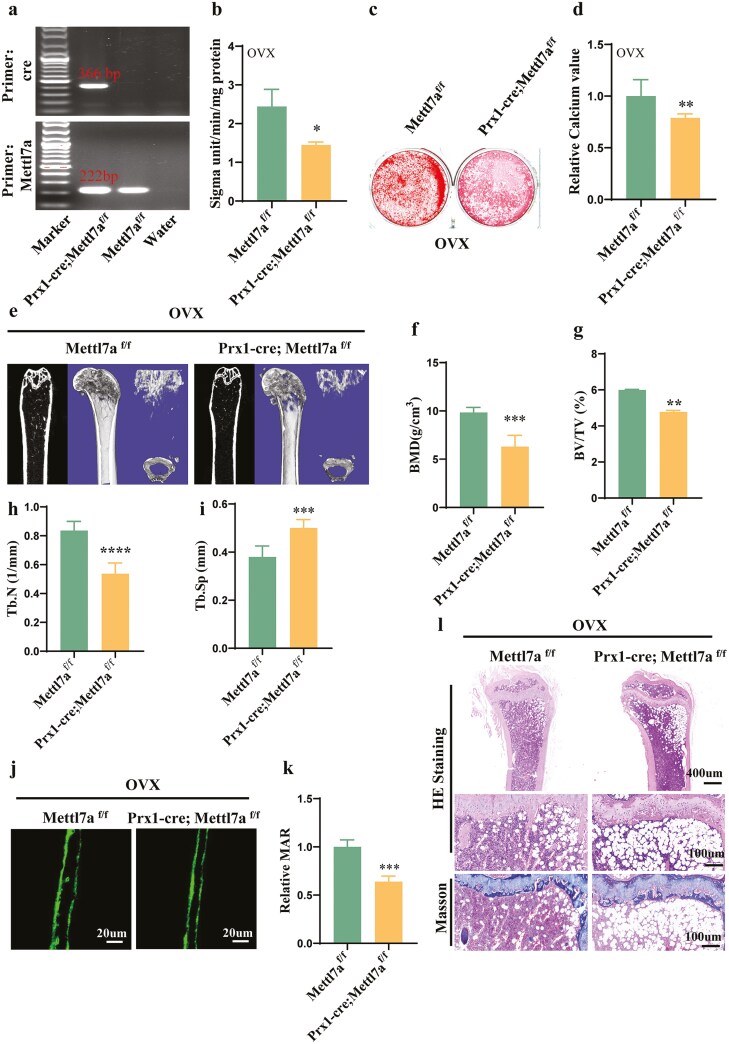
Conditional knockout of Mettl7a in mesenchyme cells accelerated bone loss of OVX mice. (A) The genotype of Mettl7a-conditional knockout mice was verified by polymerase chain reaction (PCR). (B) The ALP activity analysis in OVX-mBMSCs after 5-day osteogenic induction. (C) ARS staining after 14-day osteogenic induction in OVX-mBMSCs. (D) The relative calcium quality of (C). (E) The bone microstructure analysis by micro-CT scanning in OVX mice. (F-I) Histomorphometric quantification of femoral bone parameters, including BMD (F), BV/TV (G), Tb.N (H), and Tb.Sp (I). *n* = 7. (J-K) Representative images of calcein double staining (J) and quantitative analysis of MAR (k) of the cortical bones in OVX mice. *n* = 7. (L) HE and Masson trichrome staining of the femur of OVX mice. Scale bar = 400 μm (upper). Scale bar = 100 μm(lower). All data represent mean ± SD. Significance determined by Student’s *t*-test: *P < .05; **P < .01; ***P < .001; ****P < .0001.

Given the high overlap of Mettl7a and Prx1 expression in bone marrow, tissue-specific Mettl7a deletion in Prx1⁺ cells (CKO: Prx1-cre;Mettl7a^f/f^) was achieved by crossing Mettl7a^f/f^ mice with Prx1-Cre mice. The genotype was verified by polymerase chain reaction (Figure 2A). Next, we generated OVX model in Prx1-cre;Mettl7a^f/f^ and Mettl7a^f/f^ mice, isolated mBMSCs, and performed osteogenic differentiation induction. ALP activity assays, ARS and calcium quantification revealed significant inhibition of OVX-mBMSCs osteogenic capacity in Prx1-cre;Mettl7a^f/f^ group than Mettl7a^f/f^ group (Figure 2B-D). Bone microarchitecture alterations in OVX models were analyzed via Micro-Computed Tomography and bone histomorphometry, comparing Prx1-cre;Mettl7a^f/f^ and Mettl7a^f/f^ group. We found Mettl7a CKO significantly accelerated trabecular bone loss of OVX mice (Figure 2E). Static histomorphometric analyses revealed significant reductions of BMD, trabecular bone volume/tissue volume (BV/TV), and trabecular number (Tb.N) in Prx1-cre;Mettl7a^f/f^ mice compared to Mettl7a^f/f^ littermates (Figure 2F-H). Conversely, trabecular spacing (Tb.Sp) exhibited marked elevation in the CKO mice (Figure 2I). Calcein double-labeling confirmed diminished bone formation rates in CKO mice (Figure 2J and K). Histological evaluations (H&E/Masson staining) further demonstrated compromised trabecular architecture and increased vacuolar defects in CKO bone sections (Figure 2L).

### Mettl7a regulated the expression level and O-GlcNAcylation level of Bsp

**Figure 3.**
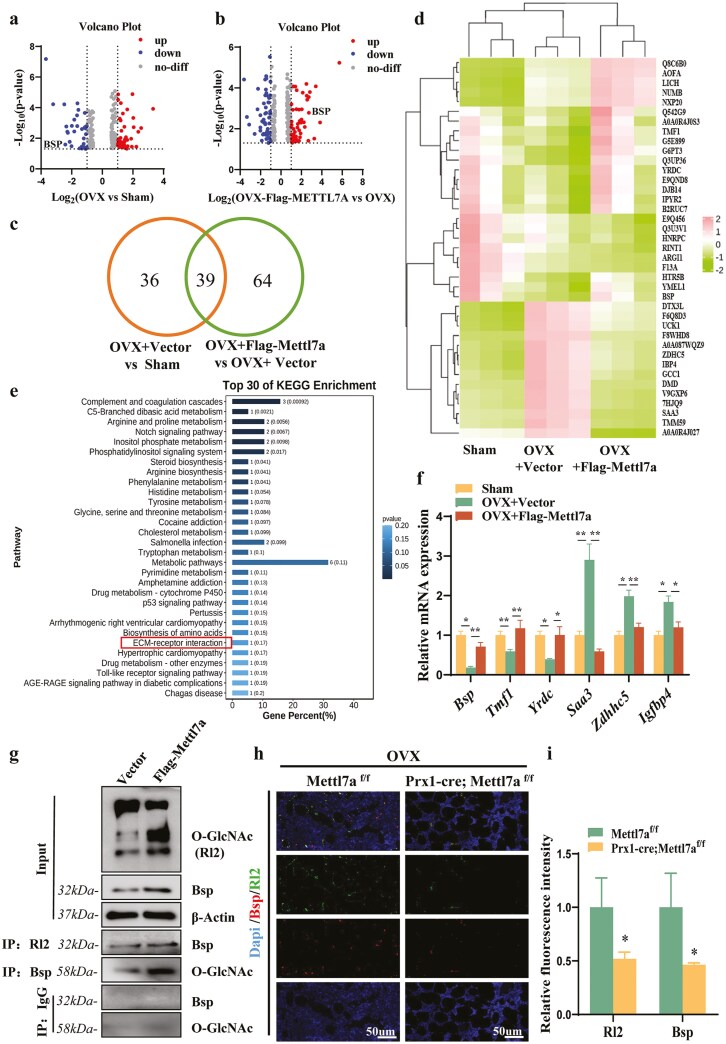
Mettl7a regulated the expression level and O-GlcNAcylation level of Bsp. (A) A volcano plot of DEPs in mBMSCs between sham and OVX + Vector group. *n* = 3. (B) A volcano plot of DEPs in mBMSCs between OVX + Vector and OVX + Flag-Mettl7a group. *n* = 3. (C) The Venn Diagram analysis of overlapping DEPs between (A) and (B), in which the DEPs are associated with osteoporosis and regulated by Mettl7a. (D) Heatmap of overlapping DEPs between (A) and (B), which are associated with osteoporosis and regulated by Mettl7a. (E) KEGG analysis of overlapping DEPs between (A) and (B), which are associated with osteoporosis and regulated by Mettl7a. (F) Proteomic analysis reliability verified by RT-qPCR of Bsp, Tmf1, Yrdc, Saa3, Zdhhc5, Igfbp4. (G) Overall O-GlcNAcylation level, overall Bsp expression level, and O-GlcNAcylated Bsp level in Vector and Flag-Mettl7a group by Co-IP. (H) Immunofluorescence of Rl2 (green), Bsp (red), and nuclei (Dapi, blue) in the femur of OVX Mettl7a ^f/f^ and Prx1-cre; Mettl7a ^f/f^ mice. Scale bar = 50μm. (I) Quantitative analysis of the relative fluorescence intensity of Rl2 and Bsp from (H). All data represent mean ± SD. Significance determined by Student’s *t*-test: *P < .05; **P < .01.

Next, we sought to identify the potential mechanism that mediate Mettl7a-regulated osteogenic differentiation using proteome analysis. mBMSCs derived from OVX and sham groups were cultured in vitro, and then overexpression Mettl7a in OVX-mBMSCs (OVX + Flag-Mettl7a). Proteomic profiling identified 75 DEPs between sham and OVX + Vector groups, with 42 proteins increased and 43 proteins decreased, respectively (Figure 3A; *P* < .05 and log2 foldchange >1 or <−1, Table S1). And there are 103 DEPs between OVX + Vector and OVX + Flag-Mettl7a group, with 51 proteins increased and 52 proteins decreased, respectively (Figure 3B; *P* < .05 and log2 foldchange > 1 or < −1). The Venn Diagram analysis revealed 39 overlapping genes between the two sets of DEPs, suggesting that Mettl7a regulated 39 core DEPs associated with osteoporosis (Figure 3C). To systematically research the osteoporosis-related DEPs regulated by Mettl7a, we generated a heatmap to visualize the 39 core DEPs (Figure 3D). KEGG analysis showed that the extracellular matrix (ECM) represented by bone sialoprotein (Bsp) was the key osteoporosis-related gene regulated by Mettl7a (Figure 3E). To validate the quantitative proteomics results, the expression of 6 DEPs including Bsp was examined (Figure 3F). Transcriptional profiles of the selected genes aligned with predicted expression patterns, indicating the reliability of our proteomic analysis. It was well known that Bsp, as one of the non-collagen proteins in the ECM, was a highly glycosylated protein. At the same time, studies have shown that O-linked N-acetylglucosaminylation (O-GlcNAcylation) may play an important role in osteogenic differentiation. Taken above, we supposed that the O-GlcNAcylation modification of Bsp may be the potential mechanism by which Mettl7a regulated osteogenic differentiation. To verify our conjecture, we next explored the effect of Mettl7a on the O-GlcNAcylation level of Bsp by Co-IP. O-GlcNAcylated protein levels were quantified via immunoblot using the O-GlcNAc antibody (Rl2). The results demonstrated that Mettl7a overexpression significantly enhanced not only the expression of Bsp and the overall O-GlcNAcylation modification level, and also the O-GlcNAcylation Bsp (Figure 3G). To further validate Mettl7a-mediated regulation of Bsp, we established an OVX model in Mettl7a CKO mice. Immunofluorescence staining showed that the expression level and O-GlcNAcylation level of Bsp in bone marrow tissues were downregulated in Prx1-cre;Mettl7a^f/f^ group (Figure 3H and I). All the above results suggest that Mettl7a-regulated Bsp O-GlcNAcylation may as a critical determinant of mBMSCs osteogenic potential.

### Mettl7a regulated osteogenic differentiation by targeting the O-GlcNAcylation of Bsp

**Figure 4.**
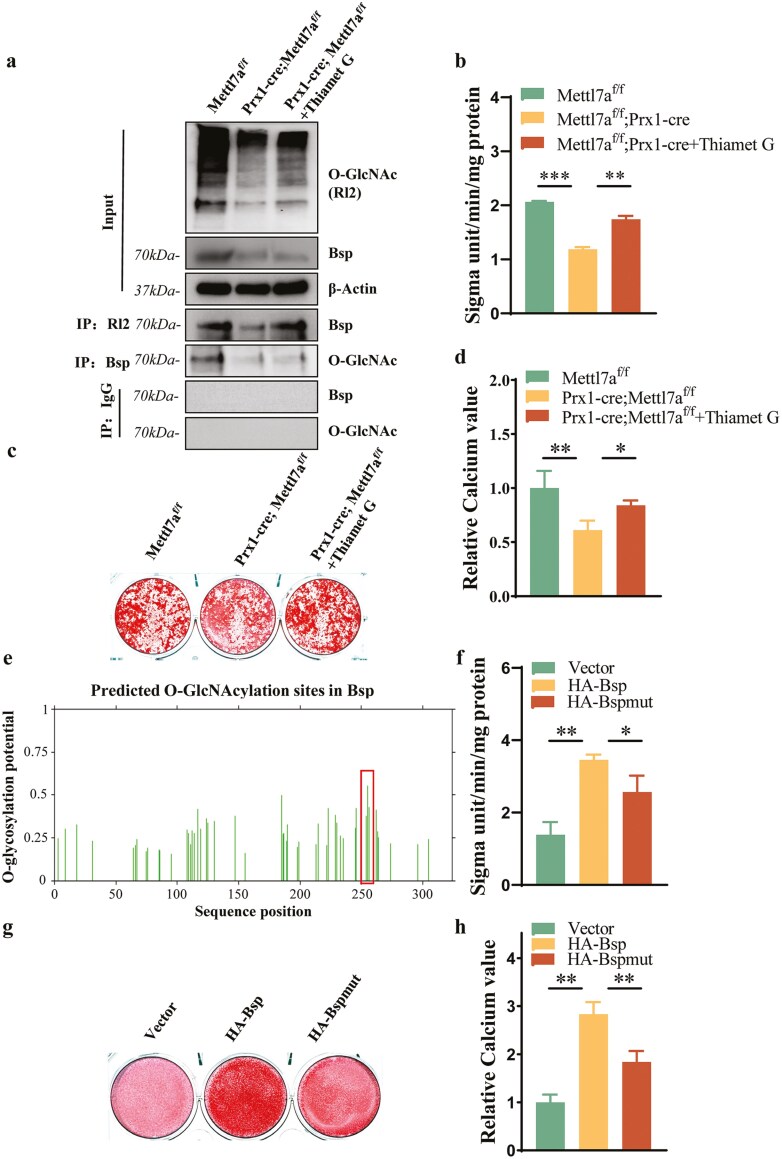
Mettl7a regulated osteogenic differentiation by targeting the O-GlcNAcylation of Bsp. (A) Co-IP analysis of overall O-GlcNAcylation level, overall Bsp expression level, and O-GlcNAcylated Bsp level in Mettl7a^f/f^, Prx1-cre;Mettl7a^f/f^ and Prx1-cre;Mettl7a^f/f^ + Thiamet G group to explore the effect of Mettl7a-conditional knockout on the endogenous interaction between the O-GlcNAcylation and Bsp. (B) The ALP activity analysis in mBMSCs after 5-day osteogenic induction. (C) ARS staining after 14-day osteogenic induction in mBMSCs. (D) The relative calcium quality of mBMSCs at induced 14 days. (E) O-GlcNAcylation sites in Bsp predicted by bioinformatic analysis website. (f) The ALP activity analysis in mBMSCs after 5-day osteogenic induction. (g) ARS staining after 14-day osteogenic induction in mBMSCs. (h) The relative calcium quality of mBMSCs at induced 14 days. All data represent mean ± SD. Significance determined by one-way ANOVA analysis: *P < .05; **P < .01; ***P < .001.

O-GlcNAcylation dynamics are enzymatically balanced by Ogt-mediated O-GlcNAc addition and Oga-catalyzed O-GlcNAc removal. To delineate Mettl7a’s modulatory role in Bsp O-GlcNAcylation, we employed the activator of O-GlcNAcylation, namely the Oga inhibitor -Thiamet G. Co-IP analysis demonstrated that Mettl7a knockout suppressed Bsp expression and its O-GlcNAc modification (Figure 4A). Among them, the O-GlcNAcylation of Bsp inhibited by the knockout of Mettl7a was rescued by adding the Thiamet G (20 uM), but the overall expression level of Bsp protein was not significantly changed, suggesting that Mettl7a did not regulate the expression of Bsp through O-GlcNAc glycosylation modification (Figure 4A). Next, the effect of O-GlcNAc glycosylation mediated by Mettl7a on the osteogenic differentiation ability of mBMSCs was detected. The ALP activity assays, ARS staining, and calcium quantification results showed the osteogenic capability of mBMSCs was significantly inhibited in Prx1-cre;Mettl7a^f/f^ group compared with Mettl7a^f/f^ group, but Thiamet G would partially rescue the inhibition of osteogenic function (Figure 4B-D). Next, we used the bioinformatic analysis website (http://www.cbs.dtu.dk/services/YinOYang/) to predict the possible Bsp O-GlcNAcylation sites. Through further analysis of biological information, we found that Bsp protein contains highly enriched O-GlcNAcylation sites (Figure 4E). Therefore, we selectively mutated the 3 highest potential O-GlcNAcylation sites at threonine (Thr) 254, 255 and Serine (Ser) 256 (254 T, 255T, 256S). The ALP activity assays, ARS staining and calcium quantification results found that the mutation of O-GlcNAcylation sites significantly inhibited the promotion effect of Bsp on osteogenic differentiation of mBMSCs in vitro (Figure 4F-H). These results confirmed that BSP promotes the osteogenic function of mBMSCs dependent on the O-GlcNAcylation, and Mettl7a regulated osteogenic differentiation by regulating the glycosylation modification of BSP protein.

### Mettl7a regulated the expression and O-GlcNAcylation of Bsp through RNA m^6^A methylation

**Figure 5.**
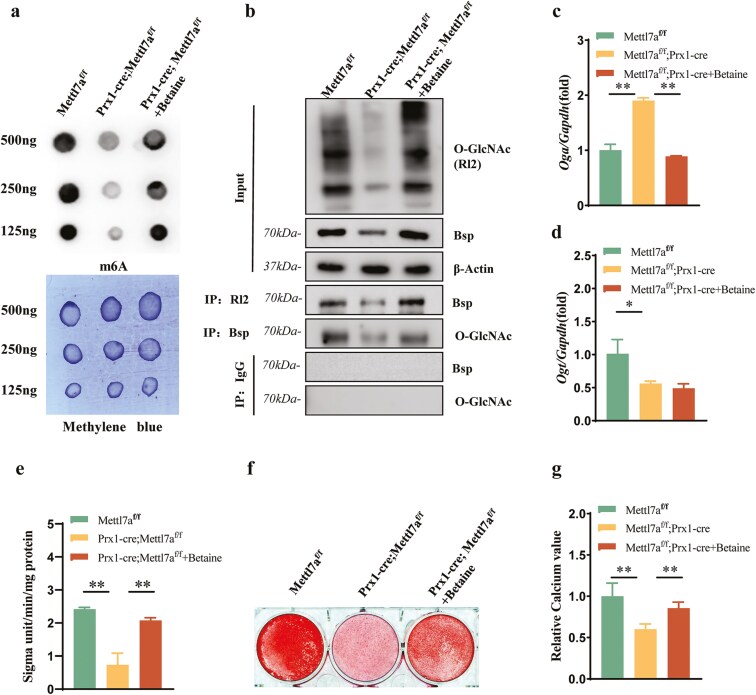
Mettl7a regulated the expression and O-GlcNAcylation of Bsp through RNA m6A methylation. (A) The effect of Mettl7a-conditional knockout on the RNA m^6^A methylation level by Dot Blot analysis. (B) Co-IP analysis of overall O-GlcNAcylation level, overall Bsp expression level and O-GlcNAcylated Bsp level in Mettl7a^f/f^, Prx1-cre;Mettl7a^f/f^ and Prx1-cre;Mettl7a^f/f^ + Betaine group to explore the role of RNA m^6^A methylation on Mettl7a mediated- interaction between the O-GlcNAcylation and Bsp. (C) Quantitative RT‒qPCR detection of the expression level of Oga. (D) Quantitative RT‒qPCR detection of the expression level of Ogt. (E) The ALP activity analysis in mBMSCs after 5-day osteogenic induction. (F) ARS staining after 14-day osteogenic induction in mBMSCs. (G) The relative calcium quality of mBMSCs at induced 14 days. All data represent mean ± SD. Significance determined by one-way ANOVA analysis: *P < .05; **P < .01; ns indicates no significance.

Based on the methyltransferase function of Mettl7a, we hypothesized that Mettl7a regulated the O-GlcNAcylation of Bsp through m^6^A methylation. Dot blot quantification revealed a marked reduction in global m^6^A levels in Prx1-cre;Mettl7a^f/f^ mice compared to the Mettl7a^f/f^ controls, and the inhibition of m^6^A level in the Prx1-cre;Mettl7a^f/f^ group could be rescued by the addition of the m^6^A activator—Betaine (Figure 5A). Co-IP results showed that the total O-GlcNAcylation level, Bsp expression level, and O-GlcNAcylation of Bsp were inhibited by Mettl7a knockout. While the addition of Betaine, a m^6^A activator, could improve the total O-GlcNAcylation level, Bsp expression level, and O-GlcNAcylation of Bsp inhibited by Mettl7a knockout (Figure 5B). Since O-GlcNAcylation was a dynamic process regulated by Ogt and Oga, we then explored the regulatory role of Mettl7a-mediated m^6^A methylation on Ogt and Oga. Real-time RT-PCR results showed that compared with the Mettl7a^f/f^ group, the expression level of *Oga* in Prx1-cre;Mettl7a^f/f^ group was increased and Ogt was decreased (Figure 5C and D). After the addition of Betaine, the increased *Oga* level was inhibited and the decreased *Ogt* level was no significant change in the Prx1-cre;Mettl7a^f/f^ group (Figure 5C and D). To investigate Mettl7a’s m^6^A-dependent regulation of osteogenesis, functional assays of ALP activity assays, ARS staining, and calcium quantification results showed that the osteogenic differentiation of mBMSCs inhibited in the Prx1-cre;Mettl7a^f/f^ group was reversed by addition of the m^6^A activator—Betaine (Figure 5E-G). In short, Mettl7a regulated the expression and O-GlcNAcylation of Bsp through the core enzymes Oga and Ogt, and m6A methylation was the key regulatory mechanism in which.

### Mettl7a-AAV treatment promoted osteogenesis and alleviated bone loss phenotype in osteoporosis mice via O-GlcNAcylation of Bsp

**Figure 6.**
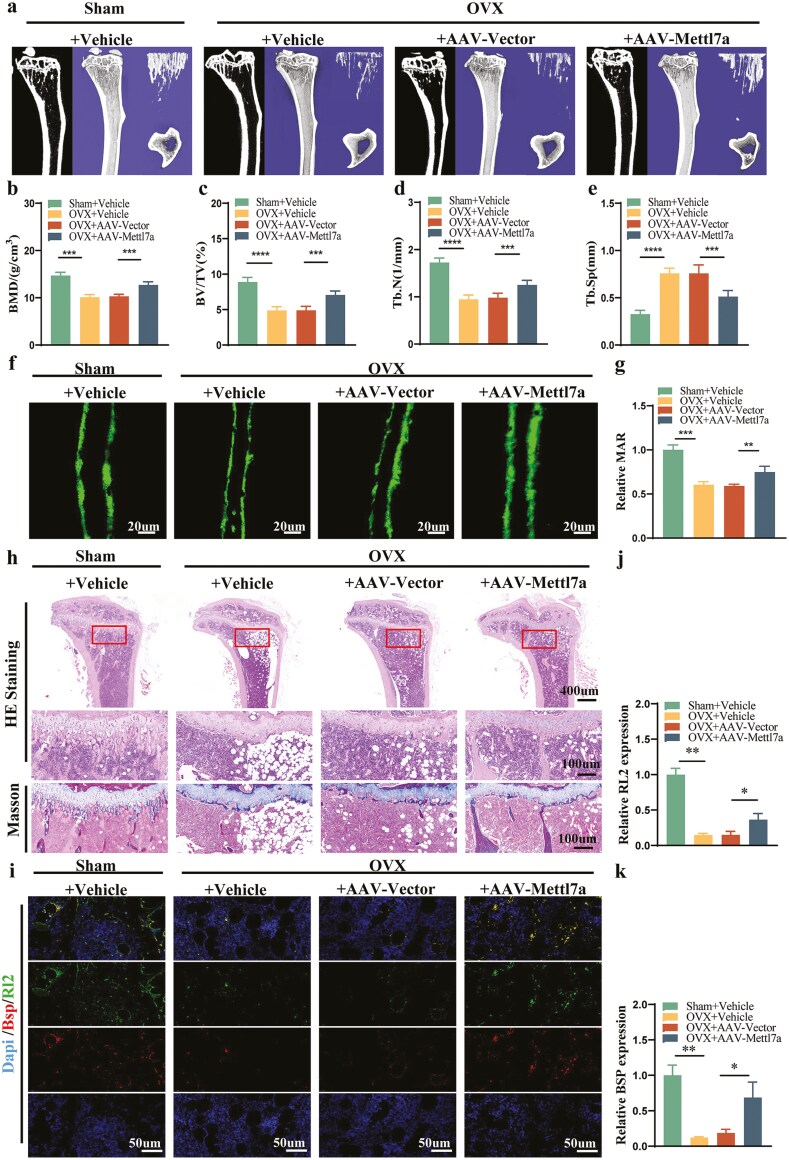
Mettl7a-AAV treatment promoted osteogenesis and alleviated bone loss phenotype in osteoporosis mice. (A) The bone microstructure analysis by micro-CT in the mice tibias. (B-E) Histomorphometric analysis of mice tibias, including BMD (b), BV/TV (C), Tb.N (D) and Tb.Sp (E). *n* = 6. (f-g) Representative images of calcein double staining (F) and quantitative analysis of relative MAR (G) of the cortical bones in mice tibias. *n* = 6. (H) HE and Masson trichrome staining of the mice tibias. Scale bar = 400 μm (upper). Scale bar = 100 μm (lower). (I) Immunofluorescence of Rl2, Bsp, and nuclei (Dapi) in the mice tibias. Scale bar = 50 μm. (J) Quantitative analysis of the Rl2 relative fluorescence intensity in (I). (K) Quantitative analysis of the Bsp relative fluorescence intensity in (I). All data represent mean ± SD. Significance determined by one-way ANOVA analysis: *P < .05; **P < .01; ***P < .001; ****P < .0001; ns indicates no significance.

**Figure 7.**
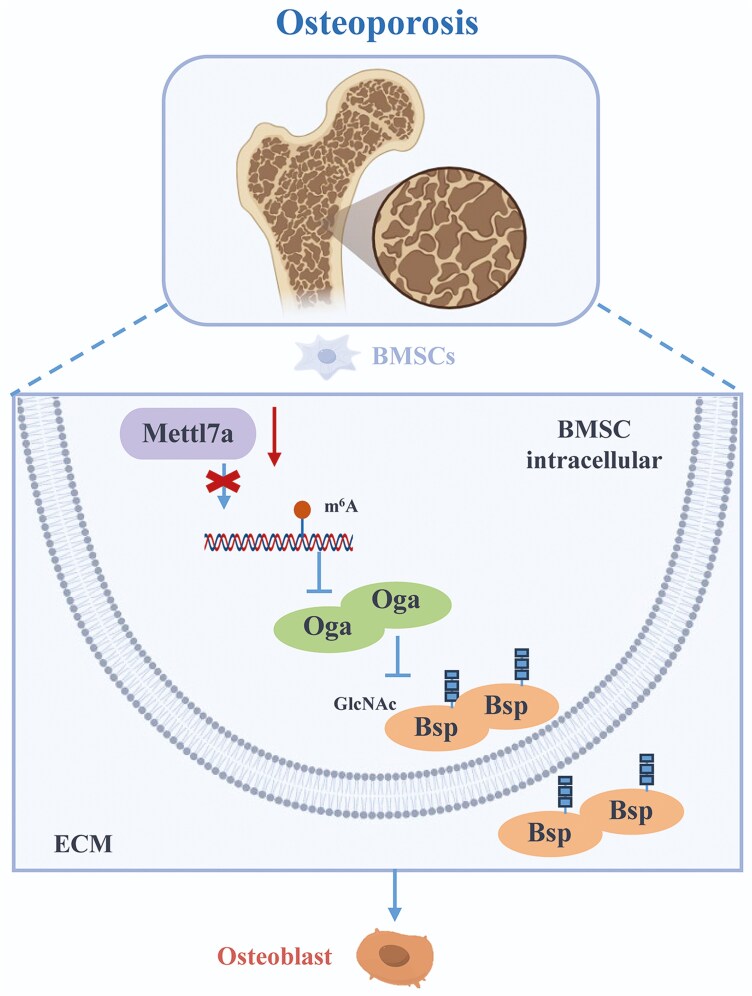
A schematic of Mettl7a in the regulation of osteoporosis by targeting the O-GlcNAcylation of Bsp via m^6^A. In the context of osteoporosis, the expression of Mettl7a was decreased, which led to the inhibition of m6A methylation within BMSCs. This suppression of methylation stimulated the expression of Oga. Subsequently, Mettl7a regulated the expression and O-GlcNAcylation of Bsp through m^6^A methylation of Oga, then promoted the differentiation of BMSCs into osteoblasts.

Given Mettl7a’s osteogenic-enhancing properties, we investigated its therapeutic potential in the OVX model via AAV9-mediated Mettl7a delivery (Figure S1A). In vivo fluorescence imaging at 14 days post-injection confirmed effective Mettl7a expression localized to the tibias (Figure S1B). Micro-CT and histomorphometric analyses revealed AAV-Mettl7a therapeutic efficacy in preventing OVX-induced bone loss (Figure 6A). The OVX mice displayed less bone mass than the sham mice, evidenced by a significant decrease in BMD, BV/TV, and Tb. N and an increase in Tb. Sp (Figure 6B-E). Furthermore, AAV-Mettl7a therapy significantly reversed the reduction in BMD, BV/TV, and Tb. N, meanwhile reduced the Tb. Sp (Figure 6B-E). Dynamic histomorphometry via calcein double labeling confirmed diminished bone formation in OVX mice, evidenced by a reduced MAR. The reductive MAR was significantly restored by AAV-Mettl7a intervention (Figure 6F and G). Histopathological assessment through H&E staining revealed less trabecular structure and bone marrow cells, meanwhile more vacuolar structure in OVX mice, but these phenotypes were partially rescued by AAV-Mettl7a therapy (Figure 6H). Masson’s trichrome staining further demonstrated compromised collagen matrix deposition and mineralization in OVX bone marrow, characterized by reduced immature collagen (blue) and mineralized collagen (red), which was also partially rescued by AAV-Mettl7a therapy (Figure 6H). Dual immunofluorescence staining demonstrated diminished expression and O-GlcNAcylation of Bsp in OVX mice, the reduction significantly attenuated by AAV-Mettl7a therapy (Figure 6I-K). These findings collectively indicated that AAV-Mettl7a alleviates bone loss phenotype while restoring Bsp O-GlcNAcylation in osteoporosis mice. Taken together, our results highlight Mettl7a as a key modulator of the interaction of Bsp protein O-GlcNAcylation with m^6^A methylation in osteoporosis (Figure 7).

## Discussion

Postmenopausal osteoporosis, a prevalent metabolic skeletal pathology, arises in part from compromised osteogenic differentiation capacity of BMSCs—a key pathogenic driver.^43^ This mechanism highlights the imperative to decode BMSC regulatory networks for osteoporosis therapy. Emerging studies implicated m^6^A modification as a critical regulator of BMSC fate determination and differentiation.^44^ Previous studies have predicted a biomarker network for identifying osteoporosis through multi-omics data integration, in which Mettl7a is one of the core differential genes.^39^ Our results demonstrated significant Mettl7a downregulation in osteoporosis mice. Moreover, Mettl7a colocalized with the expression of Prx1, a marker of BMSCs. Conditional knockout of Mettl7a in mesenchymal cells accelerated bone loss of OVX mice, suggesting the close relationship between Mettl7a with osteoporosis. Our results are consistent with those predicted by others and can serve as direct evidence for Mettl7a as an osteoporosis-related regulator. Mettl7a-mediated m^6^A modification underlay the reversing of bisphosphonates-impaired BMSCs osteogenic differentiation.^38^ Mettl7a also enhanced osteogenesis and cellular homeostasis in hBMSCs through m^6^A methylation modulation of osteogenic and survival-relative genes.^37^ Our in vitro findings demonstrated Mettl7a’s capacity to rescue osteogenic deficits in OVX-mBMSCs. Further, in vivo experiments revealed that AAV-Mettl7a treatment could alleviate bone loss phenotype and promote bone formation in OVX mice. Collectively, these data established Mettl7a as a critical modulator of BMSCs functionality and a viable therapeutic candidate for osteoporosis intervention.

Next, we sought to explore the mechanistic basis of Mettl7a-mediated bone homeostasis. Proteomics analysis filtered out ECM protein-Bsp as a key Mettl7a-mediated osteoporosis-related protein, which was significantly downregulated in OVX-mBMSCs and reversed after Mettl7a overexpression. As a member of the small integrin-binding ligand N-linked glycoprotein (SIBLING) family, BSP critically regulates skeletal development, matrix mineralization, and bone remodeling.^45^ The mature BSP knockout mice exhibited stunted growth and diminished bone turnover due to dual deficits in osteoblastic and osteoclastic activity.^46^ Knockout of Bsp impaired long bone development and compromised membranous/cortical bone matrix mineralization.^47^ Furthermore, bone marrow ablation in Bsp knockout mice triggered accelerated vascular invasion and hematopoietic reconstitution, concurrent with suppressed medullary osteogenesis.^48^ That is to say, Bsp affects the interplay between osteogenesis, osteoclastogenesis and angiogenesis. It should be noted that our study mainly focused on the role of Mettl7a and Bsp in BMSCs in the condition of osteoporosis, but not exclude possible regulatory effects on other phenotypes, like osteoclastogenesis and angiogenesis.

Moreover, our study provided the first evidence establishing Bsp O-GlcNAcylation as a critical PTM driving osteoporotic bone loss. As a highly glycosylated protein, our study revealed the fact that not only the expression but also the O-GlcNAcylation level of Bsp were significantly reduced in Prx1-cre;Mettl7a^f/f^ osteoporosis mice, and this reduction was partially rescued by AAV-Mettl7a injection. Previous studies have noted that the O-GlcNAcylation level was increased extensively during the osteogenic differentiation, and the Bsp expression was positively regulated in an O-GlcNAcylation-dependent manner.^24,49^ We further confirmed the regulatory effect of Bsp O-GlcNAcylation modification on osteogenic differentiation of mBMSCs by selectively mutating Bsp glycosylation sites. It’s worth noting that O-GlcNAcylation is closely related to multiple aspects of bone remodeling, including osteoblast-osteoclast balance, osteoblast-lipoblast balance and bone immune microenvironment.^50-52^ Therefore, additional research is necessary to thoroughly assess the influence of Bsp O-GlcNAcylation in combination with other phenotypes, in order to offer a more comprehensive comprehension of osteoporosis.

O-GlcNAcylation represents a form of dynamic PTM that is regulated by Oga and Ogt.^53,54^ In the present study, we observed that Mettl7a indirectly regulated the O-GlcNAcylation at 254 T, 255T, and 256S of Bsp by regulating the expression of Oga and Ogt, making for osteogenic differentiation. Mechanistically, the expression of Oga was mainly m^6^A dependent, but the regulatory mechanism of Ogt still needs to be further explored, since the expression of Ogt cannot be rescued by m^6^A activator-Betaine. Based on the present results, Mettl7a may indirectly regulate the O-GlcNAcylation modification of Bsp by regulating the key enzyme of O-GlcNAcylation (Oga) through m^6^A methylation. Previous studies regarding the interaction between m^6^A and O-GlcNAcylation mainly focused on the O-GlcNAc modifications of YTHDF protein, enhancing its protein stability and RNA m^6^A methylation.^27,55,56^ In this current research, we made a novel discovery that Mettl7a has the ability to modulate the protein O-GlcNAcylation via the m6A methylation of Oga. The findings offer a fresh outlook for deciphering the regulatory network of PTM.

Osteoporosis treatment focuses on preserving the bone metabolism balance and decreasing bone loss.^57^ The current strategies for treating osteoporosis mainly involve inhibiting bone resorption and promoting bone formation.^58^ Bisphosphonates (e.g., alendronate, zoledronate) serve as first-line antiresorptive agents for osteoporosis. They primarily act by inhibiting osteoclast activity and bone resorption, thereby preventing bone loss. Denosumab, a monoclonal antibody targeting RANKL, inhibits osteoclast differentiation and activity, similar to bisphosphonates. However, both of them had adverse reactions such as hypocalcemia, mandibular osteonecrosis, and atypical femoral fracture. Moreover, they both could not address the underlying issue of impaired osteoblast function. In contrast, Mettl7a-based therapy stimulates bone formation by enhancing BMSCs osteogenesis and ECM remodeling via modulation of Bsp O-GlcNAcylation. Unlike conventional antiresorptive strategies, this approach directly addresses osteoporosis’ anabolic deficit by promoting de novo bone formation. From the perspective of mechanism, the treatment of osteoporosis should not only focus on osteoblast and osteoclast function, but also the pathological microenvironment of host comorbidity is a major challenge that limits the therapeutic effect.^9,12^ Existing anabolic and antiresorptive drugs focus only on the function of osteoblasts and osteoclasts, ignoring the interplay between bone cells and ECM under the osteoporosis microenvironment. As a key highly glycosylated extracellular non-collagen protein, O-GlcNAcylation of Bsp not only promotes the osteogenic differentiation of BMSCs, but also improves ECM integrity. O-GlcNAcylation of Bsp can also bind to integrins (such as αvβ3) on the cell surface through its RGD sequence, mediating the interaction of BMSCs and osteoblasts to the extracellular matrix.^59,60^ This dual regulatory effect on BMSCs osteogenic differentiation and regenerative microenvironment makes targeting O-GlcNAcylation of Bsp therapy potentially superior to existing anti-bone resorption drugs.

It is worth noting that the Mettl7a-AAV therapy also has certain potential limitations, including potential immune responses, off-target effects, and scalability.^61-63^ Although AAV vectors are generally considered low immunogenic, there is still a risk of immune responses, particularly in patients with pre-existing immunity to AAV capsid proteins.^64^ This could limit the efficacy of Mettl7a-AAV therapy in some individuals. Besides, off-target effects of AAV-mediated gene delivery remain a concern, as unintended expression of Mettl7a in non-target tissues could lead to adverse effects.^62^ To address this, further optimization of delivery systems and tissue specificity is needed to minimize off-target effects. The scalability of AAV-based therapies is currently limited by high production costs and challenges in large-scale manufacturing. These factors could hinder the widespread clinical application of Mettl7a-AAV therapy. Future research should focus on the optimization of scalable AAV production by modifying the AAV genome or biological processes inside the cell to improve scalability.^63^

## Conclusion

In conclusion, we demonstrate that Mettl7a overexpression improved the impaired osteogenic capability of osteoporosis mBMSCs. Conditional knockout of Mettl7a in mesenchyme accelerated bone loss phenotype of osteoporosis mice. Mettl7a promoted mBMSCs osteogenic differentiation and alleviated osteoporotic bone loss by targeting O-GlcNAcylation of Bsp via m^6^A methylation. Our study provides new evidence that Mettl7a acts as a pivotal regulator and a viable therapeutic candidate for osteoporosis intervention.

## Supplementary Material

szaf024_suppl_Supplementary_Tables_S2

szaf024_suppl_Supplementary_Tables_S1

szaf024_suppl_Supplementary_Figures_S1

## Data Availability

Research data are not shared.
