# Supplementary material for: Mettl7a alleviated bone loss in osteoporosis mice by targeting the O-GlcNAcylation of Bsp via m6A methylation
Source: Stem Cells Transl Med. 2025 Jun 25;14(7):szaf024. doi: 10.1093/stcltm/szaf024 (PMC12188527; doi:10.1093/stcltm/szaf024)
Supplement: szaf024_suppl_Supplementary_Tables_S2 [file szaf024_suppl_supplementary_tables_s2.pdf]

The primers for specific genes

specific genes	Target Sequences
Gapdh-Forward	5’-GATCATCAGCAATGCCTCCT-3’
Gapdh-Reverse	5’-ACCTGGTGCTCAGTGTAGCC-3’
Mettl7a-Forward	5’-CCGGGAGGGGCTTTTTACTTC-3’
Mettl7a-Reverse	5’- CCCTGTCGATGGACTTCTGG-3’
Bsp-Forward	5’-ATGGAGACGGCGATAGTTCC-3’
Bsp-Reverse	5’-CTAGCTGTTACACCCGAGAGT-3’
Tmf1-Forward	5’- ATGAGCTGGTTCAATGCCTCG-3’
Tmf1-Reverse	5’- CCCTGTCGATGGACTTCTGG-3’
Yrdc-Forward	5’- GCCCTGAGTTGCGTGTACC-3’
Yrdc-Reverse	5’- TCACCTGACAGTACCTGTAGAC-3’
Saa3-Forward	5’- AGAGAGGCTGTTCAGAAGTTCA-3’
Saa3-Reverse	5’- AGCAGGTCGGAAGTGGTTG-3’
Zdhhc5-Forward	5’- CAAACCCAGCAAGTATGTACCG-3’
Zdhhc5-Reverse	5’- CTGGACACGTAAAGGCAAAGA-3’
Igfbp4-Forward	5’- AGAAGCCCCTGCGTACATTG-3’
Igfbp4-Reverse	5’- TTGTTGGGATGTTCGCTCTCA-3’
Oga-Forward	5’-CATAGGATGTTTTGGCGAGAGAT-3’
Oga-Reverse	5’-CCTGGCGAAATAGCATAGATGAA-3’
Ogt-Forward	5’- GACGCAACCAAACTTTGCAGT-3’
Ogt-Reverse	5’-TCAAGGGTGACAGCCTTTTCA-3’
