# Supplementary material for: Mettl7a alleviated bone loss in osteoporosis mice by targeting the O-GlcNAcylation of Bsp via m6A methylation
Source: Stem Cells Transl Med. 2025 Jun 25;14(7):szaf024. doi: 10.1093/stcltm/szaf024 (PMC12188527; doi:10.1093/stcltm/szaf024)
Supplement: szaf024_suppl_Supplementary_Tables_S1 [file szaf024_suppl_supplementary_tables_s1.pdf]

<table>
<tr><th>Row</th><th>Peak Name</th><th>Group</th><th>t-value</th><th>p-value</th><th>Fold Change</th></tr>
<tr><td>1</td><td>sp|P11531|DMD_MOUSE</td><td>Dystrophin OS=Mus musculus GN=Dmd PE=1 SV=3</td><td>-19.7559</td><td>3.87E-05</td><td>0.081621</td></tr>
<tr><td>2</td><td>tr|A2AE15|A2AE15_MOUSE</td><td>Complement factor properdin OS=Mus musculus GN=Cfp PE=1 SV=1</td><td>-6.64639</td><td>0.00266</td><td>0.098824</td></tr>
<tr><td>3</td><td>tr|Q4FK29|Q4FK29_MOUSE</td><td>C-type lectin domain family 4 member E OS=Mus musculus GN=Clec4e PE=1 SV=1</td><td>-5.25933</td><td>0.00626</td><td>0.115139</td></tr>
<tr><td>4</td><td>tr|G3UYX7|G3UYX7_MOUSE</td><td>Slit homolog 2 protein OS=Mus musculus GN=Slit2 PE=1 SV=1</td><td>-3.88024</td><td>0.01784</td><td>0.167306</td></tr>
<tr><td>5</td><td>sp|P04918|SAA3_MOUSE</td><td>Serum amyloid A-3 protein OS=Mus musculus GN=Saa3 PE=1 SV=1</td><td>-8.35951</td><td>0.00112</td><td>0.204136</td></tr>
<tr><td>6</td><td>tr|A0A0R4J027|A0A0R4J027_MOU</td><td>Protein Acod1 OS=Mus musculus GN=Acod1 PE=1 SV=1</td><td>-15.1919</td><td>0.00011</td><td>0.210339</td></tr>
<tr><td>7</td><td>sp|Q9QY73|TMM59_MOUSE</td><td>Transmembrane protein 59 OS=Mus musculus GN=Tmem59 PE=1 SV=2</td><td>-4.33328</td><td>0.01232</td><td>0.246642</td></tr>
<tr><td>8</td><td>sp|Q91V81|RBM42_MOUSE</td><td>RNA-binding protein 42 OS=Mus musculus GN=Rbm42 PE=1 SV=1</td><td>-7.01457</td><td>0.00218</td><td>0.246978</td></tr>
<tr><td>9</td><td>sp|Q8BHB4|WDR3_MOUSE</td><td>WD repeat-containing protein 3 OS=Mus musculus GN=Wdr3 PE=1 SV=1</td><td>-4.80027</td><td>0.00865</td><td>0.247883</td></tr>
<tr><td>10</td><td>tr|A2AQA7|A2AQA7_MOUSE</td><td>Intron-binding protein aquarius OS=Mus musculus GN=Aqr PE=1 SV=1</td><td>-3.04894</td><td>0.03807</td><td>0.271735</td></tr>
<tr><td>11</td><td>tr|Q542C3|Q542C3_MOUSE</td><td>Phosphorylated CTD-interacting factor 1 OS=Mus musculus GN=Pcif1 PE=1 SV=1</td><td>-5.82732</td><td>0.00432</td><td>0.28037</td></tr>
<tr><td>12</td><td>sp|P33766|FPR1_MOUSE</td><td>fMet-Leu-Phe receptor OS=Mus musculus GN=Fpr1 PE=2 SV=1</td><td>-3.79933</td><td>0.01911</td><td>0.294908</td></tr>
<tr><td>13</td><td>tr|A0A0R4J1E3|A0A0R4J1E3_MOU</td><td>Drebrin OS=Mus musculus GN=Dbn1 PE=1 SV=1</td><td>-5.70577</td><td>0.00466</td><td>0.301543</td></tr>
<tr><td>14</td><td>sp|Q9R0Q6|ARC1A_MOUSE</td><td>Actin-related protein 2/3 complex subunit 1A OS=Mus musculus GN=Arpc1a PE=1</td><td>-6.13773</td><td>0.00357</td><td>0.308341</td></tr>
<tr><td>15</td><td>tr|Q642K5|Q642K5_MOUSE</td><td>40S ribosomal protein S30 OS=Mus musculus GN=Fau PE=1 SV=1</td><td>-3.88753</td><td>0.01773</td><td>0.308424</td></tr>
<tr><td>16</td><td>sp|Q8R216|SIR4_MOUSE</td><td>NAD-dependent protein lipoamidase sirtuin-4, mitochondrial OS=Mus musculus</td><td>-9.98363</td><td>0.00057</td><td>0.312638</td></tr>
<tr><td>17</td><td>sp|P62737|ACTA_MOUSE</td><td>Actin, aortic smooth muscle OS=Mus musculus GN=Acta2 PE=1 SV=1</td><td>-3.82641</td><td>0.01868</td><td>0.325354</td></tr>
<tr><td>18</td><td>sp|O09126|SEM4D_MOUSE</td><td>Semaphorin-4D OS=Mus musculus GN=Sema4d PE=1 SV=2</td><td>-5.45534</td><td>0.00549</td><td>0.332791</td></tr>
<tr><td>19</td><td>tr|S4R1P5|S4R1P5_MOUSE</td><td>Dystonin OS=Mus musculus GN=Dst PE=1 SV=1</td><td>-5.5526</td><td>0.00515</td><td>0.337791</td></tr>
<tr><td>20</td><td>sp|P58058|NADK_MOUSE</td><td>NAD kinase OS=Mus musculus GN=Nadk PE=1 SV=2</td><td>-19.7636</td><td>3.87E-05</td><td>0.341741</td></tr>
<tr><td>21</td><td>tr|A0A0R4J2B2|A0A0R4J2B2_MOU</td><td>BTB/POZ domain-containing protein KCTD12 OS=Mus musculus GN=Kctd12 PE=1 SV=</td><td>-12.6111</td><td>0.00023</td><td>0.344374</td></tr>
<tr><td>22</td><td>sp|Q924Z5|TRAM2_MOUSE</td><td>Translocating chain-associated membrane protein 2 OS=Mus musculus GN=Tram2</td><td>-7.37072</td><td>0.00181</td><td>0.344927</td></tr>
<tr><td>23</td><td>sp|Q60972|RBBP4_MOUSE</td><td>Histone-binding protein RBBP4 OS=Mus musculus GN=Rbbp4 PE=1 SV=5</td><td>-5.31934</td><td>0.00601</td><td>0.346751</td></tr>
<tr><td>24</td><td>sp|Q62261|SPTB2_MOUSE</td><td>Spectrin beta chain, non-erythrocytic 1 OS=Mus musculus GN=Sptbn1 PE=1 SV=2</td><td>-8.82446</td><td>0.00091</td><td>0.356607</td></tr>
<tr><td>25</td><td>tr|Q569U6|Q569U6_MOUSE</td><td>Jun-B oncogene OS=Mus musculus GN=Junb PE=1 SV=1</td><td>-14.9079</td><td>0.00012</td><td>0.358848</td></tr>
<tr><td>26</td><td>tr|I7HJQ9|I7HJQ9_MOUSE</td><td>Myotubularin-related protein 1 OS=Mus musculus GN=Mtmr1 PE=1 SV=1</td><td>-21.722</td><td>2.66E-05</td><td>0.36693</td></tr>
<tr><td>27</td><td>tr|F8WHD8|F8WHD8_MOUSE</td><td>Synaptojanin-2 OS=Mus musculus GN=Synj2 PE=1 SV=1</td><td>-7.5194</td><td>0.00167</td><td>0.367232</td></tr>
<tr><td>28</td><td>tr|V9GXP6|V9GXP6_MOUSE</td><td>MARVEL domain-containing protein 1 (Fragment) OS=Mus musculus GN=Marveld1 P</td><td>-3.23443</td><td>0.03184</td><td>0.37256</td></tr>
<tr><td>29</td><td>tr|G3X8Y8|G3X8Y8_MOUSE</td><td>Toll-like receptor OS=Mus musculus GN=Tlr2 PE=1 SV=1</td><td>-20.3689</td><td>3.43E-05</td><td>0.373086</td></tr>
<tr><td>30</td><td>tr|Q4KL29|Q4KL29_MOUSE</td><td>C-type lectin domain family 4 member D OS=Mus musculus GN=Clec4d PE=1 SV=1</td><td>-3.73804</td><td>0.02016</td><td>0.373123</td></tr>
<tr><td>31</td><td>sp|Q8C0D5|EFL1_MOUSE</td><td>Elongation factor-like GTPase 1 OS=Mus musculus GN=Efl1 PE=1 SV=1</td><td>-13.3654</td><td>0.00018</td><td>0.373451</td></tr>
<tr><td>32</td><td>tr|Q80UI7|Q80UI7_MOUSE</td><td>C-type lectin domain family 4, member a1 OS=Mus musculus GN=Clec4a1 PE=1 SV</td><td>-4.04115</td><td>0.01559</td><td>0.377419</td></tr>
<tr><td>33</td><td>tr|Q3UCF2|Q3UCF2_MOUSE</td><td>Cell division cycle 5-like protein OS=Mus musculus GN=Cdc5l PE=1 SV=1</td><td>-4.23904</td><td>0.01327</td><td>0.392786</td></tr>
<tr><td>34</td><td>tr|A0A087WQB0|A0A087WQB0_MOU</td><td>Mortality factor 4-like protein 1 OS=Mus musculus GN=Morf4l1 PE=1 SV=1</td><td>-4.5743</td><td>0.01023</td><td>0.407312</td></tr>
<tr><td>35</td><td>sp|Q3URS9|CCD51_MOUSE</td><td>Coiled-coil domain-containing protein 51 OS=Mus musculus GN=Ccdc51 PE=1 SV=</td><td>-3.33774</td><td>0.0289</td><td>0.415659</td></tr>
<tr><td>36</td><td>tr|Q3TX09|Q3TX09_MOUSE</td><td>Docking protein 2 OS=Mus musculus GN=Dok2 PE=1 SV=1</td><td>-3.85347</td><td>0.01825</td><td>0.42121</td></tr>
<tr><td>37</td><td>sp|Q99104|MYO5A_MOUSE</td><td>Unconventional myosin-Va OS=Mus musculus GN=Myo5a PE=1 SV=2</td><td>-10.2528</td><td>0.00051</td><td>0.424294</td></tr>
<tr><td>38</td><td>tr|Q4FJP7|Q4FJP7_MOUSE</td><td>Monocyte differentiation antigen CD14 OS=Mus musculus GN=Cd14 PE=1 SV=1</td><td>-12.199</td><td>0.00026</td><td>0.425152</td></tr>
<tr><td>39</td><td>sp|Q9CX86|ROA0_MOUSE</td><td>Heterogeneous nuclear ribonucleoprotein A0 OS=Mus musculus GN=Hnrnpa0 PE=1</td><td>-2.77727</td><td>0.04996</td><td>0.430265</td></tr>
<tr><td>40</td><td>sp|Q9EQ80|NIF3L_MOUSE</td><td>NIF3-like protein 1 OS=Mus musculus GN=Nif3l1 PE=1 SV=4</td><td>-2.85096</td><td>0.04635</td><td>0.436228</td></tr>
<tr><td>41</td><td>sp|P16546|SPTN1_MOUSE</td><td>Spectrin alpha chain, non-erythrocytic 1 OS=Mus musculus GN=Sptan1 PE=1 SV=</td><td>-10.2179</td><td>0.00052</td><td>0.442454</td></tr>
<tr><td>42</td><td>sp|P62700|YPEL5_MOUSE</td><td>Protein yippee-like 5 OS=Mus musculus GN=Ypel5 PE=1 SV=1</td><td>-11.6625</td><td>0.00031</td><td>0.445295</td></tr>
<tr><td>43</td><td>sp|P60670|NPL4_MOUSE</td><td>Nuclear protein localization protein 4 homolog OS=Mus musculus GN=Nploc4 PE</td><td>-6.50696</td><td>0.00288</td><td>0.447836</td></tr>
<tr><td>44</td><td>tr|Q3U432|Q3U432_MOUSE</td><td>Growth arrest-specific protein 7 OS=Mus musculus GN=Gas7 PE=1 SV=1</td><td>-5.46151</td><td>0.00546</td><td>0.452146</td></tr>
<tr><td>45</td><td>sp|Q9CQU1|MFAP1_MOUSE</td><td>Microfibrillar-associated protein 1 OS=Mus musculus GN=Mfap1 PE=1 SV=1</td><td>-3.39861</td><td>0.02731</td><td>0.45438</td></tr>
<tr><td>46</td><td>tr|J3QNK5|J3QNK5_MOUSE</td><td>Histone deacetylase complex subunit SAP130 OS=Mus musculus GN=Sap130 PE=1 S</td><td>-4.03804</td><td>0.01563</td><td>0.470979</td></tr>
<tr><td>47</td><td>tr|Q6W4W7|Q6W4W7_MOUSE</td><td>DIA3 OS=Mus musculus GN=Diaph2 PE=1 SV=1</td><td>-37.5537</td><td>3.00E-06</td><td>0.471886</td></tr>
<tr><td>48</td><td>sp|Q924A2|CIC_MOUSE</td><td>Protein capicua homolog OS=Mus musculus GN=Cic PE=1 SV=2</td><td>-7.31502</td><td>0.00186</td><td>0.477746</td></tr>
<tr><td>49</td><td>tr|Q8C845|Q8C845_MOUSE</td><td>EF-hand domain-containing protein D2 OS=Mus musculus GN=Efhd2 PE=1 SV=1</td><td>-12.5157</td><td>0.00023</td><td>0.480301</td></tr>
<tr><td>50</td><td>sp|Q9D9H8|CB069_MOUSE</td><td>UPF0565 protein C2orf69 homolog OS=Mus musculus PE=2 SV=3</td><td>-7.84871</td><td>0.00142</td><td>0.481485</td></tr>
<tr><td>51</td><td>sp|Q61176|ARGI1_MOUSE</td><td>Arginase-1 OS=Mus musculus GN=Arg1 PE=1 SV=1</td><td>-18.5695</td><td>4.95E-05</td><td>0.489962</td></tr>
<tr><td>52</td><td>sp|Q8VCH2|CLM5_MOUSE</td><td>CMRF35-like molecule 5 OS=Mus musculus GN=Cd300ld PE=1 SV=1</td><td>-3.00511</td><td>0.03974</td><td>0.497727</td></tr>
<tr><td>53</td><td>tr|Q3UJ34|Q3UJ34_MOUSE</td><td>Argininosuccinate synthase OS=Mus musculus GN=Ass1 PE=1 SV=1</td><td>-21.4021</td><td>2.82E-05</td><td>0.502606</td></tr>
<tr><td>54</td><td>sp|P30416|FKBP4_MOUSE</td><td>Peptidyl-prolyl cis-trans isomerase FKBP4 OS=Mus musculus GN=Fkbp4 PE=1 SV=</td><td>-3.82037</td><td>0.01877</td><td>0.508323</td></tr>
<tr><td>55</td><td>sp|Q6ZPT1|KLHL9_MOUSE</td><td>Kelch-like protein 9 OS=Mus musculus GN=Klhl9 PE=2 SV=2</td><td>-3.19294</td><td>0.03312</td><td>0.517326</td></tr>
<tr><td>56</td><td>sp|Q9Z204|HNRPC_MOUSE</td><td>Heterogeneous nuclear ribonucleoproteins C1/C2 OS=Mus musculus GN=Hnrnpc PE</td><td>-4.59555</td><td>0.01006</td><td>0.525068</td></tr>
<tr><td>57</td><td>sp|P28033|CEBPB_MOUSE</td><td>CCAAT/enhancer-binding protein beta OS=Mus musculus GN=Cebpb PE=1 SV=1</td><td>-3.15855</td><td>0.03423</td><td>0.529298</td></tr>
<tr><td>58</td><td>tr|Q3U3V1|Q3U3V1_MOUSE</td><td>Coagulation factor X OS=Mus musculus GN=F10 PE=1 SV=1</td><td>-7.29608</td><td>0.00188</td><td>0.531669</td></tr>
<tr><td>59</td><td>sp|Q8BZ36|RINT1_MOUSE</td><td>RAD50-interacting protein 1 OS=Mus musculus GN=Rint1 PE=1 SV=2</td><td>-28.3865</td><td>9.16E-06</td><td>0.534335</td></tr>
<tr><td>60</td><td>sp|Q7TT37|ELP1_MOUSE</td><td>Elongator complex protein 1 OS=Mus musculus GN=Ikbkap PE=1 SV=2</td><td>-3.72155</td><td>0.02045</td><td>0.536962</td></tr>
<tr><td>61</td><td>sp|Q8BH61|F13A_MOUSE</td><td>Coagulation factor XIII A chain OS=Mus musculus GN=F13a1 PE=1 SV=3</td><td>-3.68764</td><td>0.02106</td><td>0.537891</td></tr>
<tr><td>62</td><td>tr|A0A0G2JEX1|A0A0G2JEX1_MOU</td><td>Nexilin OS=Mus musculus GN=Nexn PE=1 SV=1</td><td>-11.9234</td><td>0.00028</td><td>0.539514</td></tr>
<tr><td>63</td><td>tr|A0A087WQZ9|A0A087WQZ9_MOU</td><td>Lymphocyte antigen 96 OS=Mus musculus GN=Ly96 PE=1 SV=1</td><td>-6.22826</td><td>0.00338</td><td>0.542178</td></tr>
<tr><td>64</td><td>sp|E9Q784|ZC3HD_MOUSE</td><td>Zinc finger CCCH domain-containing protein 13 OS=Mus musculus GN=Zc3h13 PE=</td><td>-2.92523</td><td>0.04302</td><td>0.552372</td></tr>
<tr><td>65</td><td>sp|Q3U9N9|MOT10_MOUSE</td><td>Monocarboxylate transporter 10 OS=Mus musculus GN=Slc16a10 PE=1 SV=1</td><td>-5.86053</td><td>0.00423</td><td>0.553978</td></tr>
<tr><td>66</td><td>tr|A0A0R4J170|A0A0R4J170_MOU</td><td>Transcription activator BRG1 OS=Mus musculus GN=Smarca4 PE=1 SV=1</td><td>-3.96737</td><td>0.01658</td><td>0.557834</td></tr>
<tr><td>67</td><td>sp|Q60605|MYL6_MOUSE</td><td>Myosin light polypeptide 6 OS=Mus musculus GN=Myl6 PE=1 SV=3</td><td>-8.68229</td><td>0.00097</td><td>0.559058</td></tr>
<tr><td>68</td><td>sp|Q9Z1R3|APOM_MOUSE</td><td>Apolipoprotein M OS=Mus musculus GN=Apom PE=1 SV=1</td><td>-12.2813</td><td>0.00025</td><td>0.571364</td></tr>
<tr><td>69</td><td>tr|D3YZ08|D3YZ08_MOUSE</td><td>Telomeric repeat-binding factor 2 OS=Mus musculus GN=Terf2 PE=1 SV=1</td><td>-4.60033</td><td>0.01003</td><td>0.573244</td></tr>
<tr><td>70</td><td>sp|Q8BTI8|SRRM2_MOUSE</td><td>Serine/arginine repetitive matrix protein 2 OS=Mus musculus GN=Srrm2 PE=1 S</td><td>-5.07311</td><td>0.00711</td><td>0.584923</td></tr>
<tr><td>71</td><td>tr|B1AVH5|B1AVH5_MOUSE</td><td>Coronin OS=Mus musculus GN=Coro2a PE=1 SV=1</td><td>-9.01328</td><td>0.00084</td><td>0.59333</td></tr>
<tr><td>72</td><td>sp|Q60598|SRC8_MOUSE</td><td>Src substrate cortactin OS=Mus musculus GN=Cttn PE=1 SV=2</td><td>-6.08657</td><td>0.00368</td><td>0.594687</td></tr>
<tr><td>73</td><td>sp|Q9Z2T6|KRT85_MOUSE</td><td>Keratin, type II cuticular Hb5 OS=Mus musculus GN=Krt85 PE=1 SV=2</td><td>-9.48988</td><td>0.00069</td><td>0.605915</td></tr>
<tr><td>74</td><td>sp|Q8VDZ4|ZDHC5_MOUSE</td><td>Palmitoyltransferase ZDHHC5 OS=Mus musculus GN=Zdhhc5 PE=1 SV=1</td><td>-4.95544</td><td>0.00773</td><td>0.606714</td></tr>
<tr><td>75</td><td>sp|O35298|AOAH_MOUSE</td><td>Acyloxyacyl hydrolase OS=Mus musculus GN=Aoah PE=2 SV=1</td><td>-4.9238</td><td>0.00791</td><td>0.607603</td></tr>
<tr><td>76</td><td>sp|Q05CL8|LARP7_MOUSE</td><td>La-related protein 7 OS=Mus musculus GN=Larp7 PE=1 SV=2</td><td>-3.21678</td><td>0.03238</td><td>0.610459</td></tr>
<tr><td>77</td><td>sp|Q9D4H2|GCC1_MOUSE</td><td>GRIP and coiled-coil domain-containing protein 1 OS=Mus musculus GN=Gcc1 PE</td><td>-5.07884</td><td>0.00709</td><td>0.611066</td></tr>
<tr><td>78</td><td>tr|A1L3B8|A1L3B8_MOUSE</td><td>26S proteasome non-ATPase regulatory subunit 7 OS=Mus musculus GN=Psmd7 PE=</td><td>-15.2111</td><td>0.00011</td><td>0.612822</td></tr>
<tr><td>79</td><td>tr|Q6L8F5|Q6L8F5_MOUSE</td><td>BHLH transcriptional factor Dec2 OS=Mus musculus GN=Bhlhe41 PE=1 SV=1</td><td>-15.1041</td><td>0.00011</td><td>0.61871</td></tr>
<tr><td>80</td><td>sp|P62071|RRAS2_MOUSE</td><td>Ras-related protein R-Ras2 OS=Mus musculus GN=Rras2 PE=1 SV=1</td><td>-4.23392</td><td>0.01333</td><td>0.619336</td></tr>
<tr><td>81</td><td>tr|E9Q456|E9Q456_MOUSE</td><td>Tropomyosin alpha-1 chain OS=Mus musculus GN=Tpm1 PE=1 SV=1</td><td>-3.47165</td><td>0.02555</td><td>0.622526</td></tr>
<tr><td>82</td><td>tr|Q3TLX9|Q3TLX9_MOUSE</td><td>Acid sphingomyelinase-like phosphodiesterase 3b OS=Mus musculus GN=Smpdl3b</td><td>-7.02317</td><td>0.00217</td><td>0.626549</td></tr>
<tr><td>83</td><td>tr|F6Q8D3|F6Q8D3_MOUSE</td><td>Phospholipid-transporting ATPase OS=Mus musculus GN=Atp11c PE=1 SV=1</td><td>-8.98168</td><td>0.00085</td><td>0.629957</td></tr>
<tr><td>84</td><td>tr|Q923C7|Q923C7_MOUSE</td><td>C-type lectin domain family 4 member A OS=Mus musculus GN=Clec4a2 PE=1 SV=1</td><td>-10.984</td><td>0.00039</td><td>0.634597</td></tr>
<tr><td>85</td><td>sp|P47879|IBP4_MOUSE</td><td>Insulin-like growth factor-binding protein 4 OS=Mus musculus GN=Igfbp4 PE=1</td><td>-4.06141</td><td>0.01533</td><td>0.637549</td></tr>
<tr><td>86</td><td>sp|O89001|CBPD_MOUSE</td><td>Carboxypeptidase D OS=Mus musculus GN=Cpd PE=1 SV=2</td><td>-12.2832</td><td>0.00025</td><td>0.639921</td></tr>
<tr><td>87</td><td>tr|E9QN31|E9QN31_MOUSE</td><td>Probable 28S rRNA (cytosine-C(5))-methyltransferase OS=Mus musculus GN=Nop2</td><td>-4.67491</td><td>0.00948</td><td>0.641244</td></tr>
<tr><td>88</td><td>sp|Q9CQ71|RFA3_MOUSE</td><td>Replication protein A 14 kDa subunit OS=Mus musculus GN=Rpa3 PE=1 SV=1</td><td>-5.811</td><td>0.00436</td><td>0.641309</td></tr>
<tr><td>89</td><td>sp|P01027|CO3_MOUSE</td><td>Complement C3 OS=Mus musculus GN=C3 PE=1 SV=3</td><td>-12.4213</td><td>0.00024</td><td>0.645225</td></tr>
</table>

90	sp|P58742|AAAS_MOUSE	Aladin OS=Mus musculus GN=Aaas PE=1 SV=1	-10.0301	0.00056	0.64687
91	tr|Q5SWT9|Q5SWT9_MOUSE	DNA-binding protein Ikaros OS=Mus musculus GN=Ikzf1 PE=1 SV=1	-5.66961	0.00477	0.647333
92	sp|P52623|UCK1_MOUSE	Uridine-cytidine kinase 1 OS=Mus musculus GN=Uck1 PE=1 SV=2	-6.07389	0.00371	0.648508
93	tr|E9Q0Y4|E9Q0Y4_MOUSE	Signal-induced proliferation-associated protein 1 OS=Mus musculus GN=Sipa1	-4.51197	0.01072	0.650625
94	tr|Q543J5|Q543J5_MOUSE	Antithrombin OS=Mus musculus GN=Serpinc1 PE=1 SV=1	-10.5582	0.00046	0.653868
95	sp|P49710|HCLS1_MOUSE	Hematopoietic lineage cell-specific protein OS=Mus musculus GN=Hcls1 PE=1 S	-7.17383	0.002	0.654381
96	sp|Q99JB2|STML2_MOUSE	Stomatin-like protein 2, mitochondrial OS=Mus musculus GN=Stoml2 PE=1 SV=1	-3.12592	0.03532	0.656339
97	sp|Q8C0E3|TRI47_MOUSE	Tripartite motif-containing protein 47 OS=Mus musculus GN=Trim47 PE=1 SV=2	-9.91387	0.00058	0.656856
98	sp|Q99LE1|RIPL2_MOUSE	RILP-like protein 2 OS=Mus musculus GN=Rilpl2 PE=1 SV=1	-3.58509	0.02306	0.657147
99	sp|P97298|PEDF_MOUSE	Pigment epithelium-derived factor OS=Mus musculus GN=Serpinf1 PE=1 SV=2	-7.1681	0.00201	0.658908
100	tr|Q5F2A7|Q5F2A7_MOUSE	Eukaryotic initiation factor 4A-I OS=Mus musculus GN=Eif4a1 PE=1 SV=1	-16.3312	8.23E-05	0.663212
101	tr|A0A0R4IZW6|A0A0R4IZW6_MOU	Differentially-expressed in FDCP 8 OS=Mus musculus GN=Def8 PE=1 SV=1	-6.84398	0.00239	0.663712
102	tr|D3Z795|D3Z795_MOUSE	Proteasome assembly chaperone 1 OS=Mus musculus GN=Psmg1 PE=1 SV=1	-4.16422	0.0141	0.664711
103	tr|E9Q7G0|E9Q7G0_MOUSE	Protein Numa1 OS=Mus musculus GN=Numa1 PE=1 SV=1	-7.99231	0.00133	0.665436
104	sp|Q3UIR3|DTX3L_MOUSE	E3 ubiquitin-protein ligase DTX3L OS=Mus musculus GN=Dtx3l PE=1 SV=1	-7.59647	0.00161	0.66589
105	sp|Q8JZV7|NAGA_MOUSE	N-acetylglucosamine-6-phosphate deacetylase OS=Mus musculus GN=Amdhd2 PE=1	9.297451	0.00074	1.499618
106	sp|P97429|ANXA4_MOUSE	Annexin A4 OS=Mus musculus GN=Anxa4 PE=1 SV=4	10.01311	0.00056	1.501017
107	sp|Q9R0Q7|TEBP_MOUSE	Prostaglandin E synthase 3 OS=Mus musculus GN=Ptges3 PE=1 SV=1	5.567931	0.0051	1.507965
108	tr|E9PYB0|E9PYB0_MOUSE	Protein Ahnak2 (Fragment) OS=Mus musculus GN=Ahnak2 PE=1 SV=7	7.490184	0.0017	1.513415
109	tr|Q5EBQ2|Q5EBQ2_MOUSE	MCG7941, isoform CRA_f OS=Mus musculus GN=Pebp1 PE=1 SV=1	8.195151	0.00121	1.516035
110	tr|E9QA15|E9QA15_MOUSE	Protein Cald1 OS=Mus musculus GN=Cald1 PE=1 SV=1	7.635351	0.00158	1.517202
111	sp|P97449|AMPN_MOUSE	Aminopeptidase N OS=Mus musculus GN=Anpep PE=1 SV=4	20.38495	3.42E-05	1.517954
112	sp|Q64133|AOFA_MOUSE	Amine oxidase [flavin-containing] A OS=Mus musculus GN=Maoa PE=1 SV=3	11.84521	0.00029	1.519112
113	sp|Q8CHQ0|FBX4_MOUSE	F-box only protein 4 OS=Mus musculus GN=Fbxo4 PE=1 SV=2	6.494985	0.0029	1.528256
114	sp|A2AIV8|CARD9_MOUSE	Caspase recruitment domain-containing protein 9 OS=Mus musculus GN=Card9 PE	13.71235	0.00016	1.529091
115	tr|Q790Y8|Q790Y8_MOUSE	Glucose-6-phosphate 1-dehydrogenase OS=Mus musculus GN=G6pdx PE=1 SV=1	9.944406	0.00057	1.529948
116	tr|Q5M8R8|Q5M8R8_MOUSE	60S acidic ribosomal protein P0 OS=Mus musculus GN=Rplp0 PE=1 SV=1	4.238089	0.01328	1.53841
117	sp|P14602|HSPB1_MOUSE	Heat shock protein beta-1 OS=Mus musculus GN=Hspb1 PE=1 SV=3	13.36347	0.00018	1.539072
118	sp|Q9ER72|SYCC_MOUSE	Cysteine--tRNA ligase, cytoplasmic OS=Mus musculus GN=Cars PE=1 SV=2	7.512901	0.00168	1.541305
119	sp|Q8BT60|CPNE3_MOUSE	Copine-3 OS=Mus musculus GN=Cpne3 PE=1 SV=2	6.632674	0.00268	1.544111
120	sp|O35640|ANXA8_MOUSE	Annexin A8 OS=Mus musculus GN=Anxa8 PE=1 SV=2	3.26109	0.03105	1.546032
121	sp|O09117|SYPL1_MOUSE	Synaptophysin-like protein 1 OS=Mus musculus GN=Sypl1 PE=1 SV=2	12.37554	0.00025	1.548721
122	tr|Q542H7|Q542H7_MOUSE	Fabp4 protein OS=Mus musculus GN=Fabp4 PE=1 SV=1	22.8898	2.16E-05	1.549473
123	sp|Q9DBY8|NVL_MOUSE	Nuclear valosin-containing protein-like OS=Mus musculus GN=Nvl PE=1 SV=1	3.935494	0.01702	1.551385
124	tr|F7DBB3|F7DBB3_MOUSE	Protein Ahnak2 (Fragment) OS=Mus musculus GN=Ahnak2 PE=1 SV=1	4.190216	0.0138	1.552586
125	tr|A0A0R4J055|A0A0R4J055_MOU	Dynamin-binding protein OS=Mus musculus GN=Dnmbp PE=1 SV=1	4.008185	0.01602	1.553061
126	tr|Q3UCD9|Q3UCD9_MOUSE	Cathepsin D OS=Mus musculus GN=Ctsd PE=1 SV=1	11.05071	0.00038	1.557276
127	sp|Q9QZS3|NUMB_MOUSE	Protein numb homolog OS=Mus musculus GN=Numb PE=1 SV=1	4.069645	0.01523	1.564053
128	sp|Q9R069|BCAM_MOUSE	Basal cell adhesion molecule OS=Mus musculus GN=Bcam PE=1 SV=1	15.72563	9.55E-05	1.568625
129	sp|Q9CQA5|MED4_MOUSE	Mediator of RNA polymerase II transcription subunit 4 OS=Mus musculus GN=Me	8.91677	0.00087	1.569406
130	sp|Q99N87|RT05_MOUSE	28S ribosomal protein S5, mitochondrial OS=Mus musculus GN=Mrps5 PE=1 SV=1	4.87354	0.0082	1.583816
131	sp|Q920A5|RISC_MOUSE	Retinoid-inducible serine carboxypeptidase OS=Mus musculus GN=Scpep1 PE=1 S	5.678922	0.00475	1.591016
132	sp|B2RXV4|FLVC1_MOUSE	Feline leukemia virus subgroup C receptor-related protein 1 OS=Mus musculus	4.264249	0.01301	1.595186
133	sp|Q9DD03|RAB13_MOUSE	Ras-related protein Rab-13 OS=Mus musculus GN=Rab13 PE=1 SV=1	5.09715	0.007	1.597309
134	sp|P29268|CTGF_MOUSE	Connective tissue growth factor OS=Mus musculus GN=Ctgf PE=2 SV=3	10.46547	0.00047	1.598416
135	sp|Q99KI3|EMC3_MOUSE	ER membrane protein complex subunit 3 OS=Mus musculus GN=Emc3 PE=1 SV=3	10.74031	0.00043	1.604135
136	sp|Q9Z0M5|LICH_MOUSE	Lysosomal acid lipase/cholesteryl ester hydrolase OS=Mus musculus GN=Lipa P	5.451437	0.0055	1.60997
137	sp|Q91VM9|IPYR2_MOUSE	Inorganic pyrophosphatase 2, mitochondrial OS=Mus musculus GN=Ppa2 PE=1 SV=	5.551802	0.00515	1.61778
138	tr|E9QJS1|E9QJS1_MOUSE	Non-receptor tyrosine-protein kinase TYK2 OS=Mus musculus GN=Tyk2 PE=1 SV=1	9.06856	0.00082	1.618603
139	sp|Q80SY3|VA0D2_MOUSE	V-type proton ATPase subunit d 2 OS=Mus musculus GN=Atp6v0d2 PE=2 SV=2	7.318001	0.00186	1.622433
140	sp|P61967|AP1S1_MOUSE	AP-1 complex subunit sigma-1A OS=Mus musculus GN=Ap1s1 PE=1 SV=1	3.013885	0.0394	1.625943
141	tr|A0A0R4J1C8|A0A0R4J1C8_MOU	Macrosialin OS=Mus musculus GN=Cd68 PE=1 SV=1	10.57074	0.00045	1.631855
142	tr|Q497I3|Q497I3_MOUSE	Fatty acid binding protein 5, epidermal OS=Mus musculus GN=Fabp5 PE=1 SV=1	12.44313	0.00024	1.647457
143	sp|Q8BTY8|SCFD2_MOUSE	Sec1 family domain-containing protein 2 OS=Mus musculus GN=Scfd2 PE=1 SV=1	12.35071	0.00025	1.654032
144	sp|Q02248|CTNB1_MOUSE	Catenin beta-1 OS=Mus musculus GN=Ctnnb1 PE=1 SV=1	5.640288	0.00486	1.664981
145	tr|Q8C6B0|Q8C6B0_MOUSE	MCG20149, isoform CRA_a OS=Mus musculus GN=Mettl7a1 PE=1 SV=1	8.466741	0.00107	1.671787
146	tr|B2RUC7|B2RUC7_MOUSE	Serine-threonine kinase receptor-associated protein OS=Mus musculus GN=Stra	9.435223	0.0007	1.680775
147	sp|P56213|ALR_MOUSE	FAD-linked sulfhydryl oxidase ALR OS=Mus musculus GN=Gfer PE=1 SV=2	4.417325	0.01153	1.690569
148	sp|Q8BMD8|SCMC1_MOUSE	Calcium-binding mitochondrial carrier protein SCaMC-1 OS=Mus musculus GN=Sl	8.435224	0.00108	1.692734
149	sp|Q9CQJ8|NDUB9_MOUSE	NADH dehydrogenase [ubiquinone] 1 beta subcomplex subunit 9 OS=Mus musculus	3.21565	0.03241	1.697984
150	sp|P06801|MAOX_MOUSE	NADP-dependent malic enzyme OS=Mus musculus GN=Me1 PE=1 SV=2	3.632912	0.0221	1.699077
151	sp|Q8BXA5|CLP1L_MOUSE	Cleft lip and palate transmembrane protein 1-like protein OS=Mus musculus G	9.168836	0.00079	1.699648
152	tr|G3UVW1|G3UVW1_MOUSE	Mpv17 transgene, kidney disease mutant, isoform CRA_b OS=Mus musculus GN=Mp	3.941089	0.01694	1.699758
153	sp|Q9EQ20|MMSA_MOUSE	Methylmalonate-semialdehyde dehydrogenase [acylating], mitochondrial OS=Mus	6.082517	0.00369	1.705546
154	tr|Q14B01|Q14B01_MOUSE	Protein Rnf113a2 OS=Mus musculus GN=Rnf113a2 PE=1 SV=1	3.61013	0.02255	1.707268
155	sp|Q3UKJ7|SMU1_MOUSE	WD40 repeat-containing protein SMU1 OS=Mus musculus GN=Smu1 PE=2 SV=2	3.033518	0.03865	1.708127
156	tr|A0A0R4J1D0|A0A0R4J1D0_MOU	Copine-2 OS=Mus musculus GN=Cpne2 PE=1 SV=1	15.98035	8.97E-05	1.72194
157	sp|Q91WC9|DGLB_MOUSE	Sn1-specific diacylglycerol lipase beta OS=Mus musculus GN=Daglb PE=1 SV=2	3.11515	0.03569	1.731769
158	sp|Q9CZB0|C560_MOUSE	Succinate dehydrogenase cytochrome b560 subunit, mitochondrial OS=Mus muscu	8.420653	0.00109	1.732074
159	sp|Q9CR68|UCRI_MOUSE	Cytochrome b-c1 complex subunit Rieske, mitochondrial OS=Mus musculus GN=Uq	7.422118	0.00176	1.739314
160	tr|F8WI35|F8WI35_MOUSE	Histone H3 OS=Mus musculus GN=H3f3a PE=3 SV=1	4.513495	0.01071	1.747818
161	tr|Q91XH5|Q91XH5_MOUSE	Sepiapterin reductase OS=Mus musculus GN=Spr PE=1 SV=1	6.748583	0.00251	1.752061
162	sp|Q9D281|NXP20_MOUSE	Protein Noxp20 OS=Mus musculus GN=Fam114a1 PE=1 SV=1	4.596853	0.01005	1.755098
163	sp|Q9CXD6|MCUR1_MOUSE	Mitochondrial calcium uniporter regulator 1 OS=Mus musculus GN=Mcur1 PE=1 S	3.876746	0.01789	1.767355
164	sp|Q149L6|DJB14_MOUSE	DnaJ homolog subfamily B member 14 OS=Mus musculus GN=Dnajb14 PE=2 SV=1	3.902619	0.0175	1.768957
165	sp|O09167|RL21_MOUSE	60S ribosomal protein L21 OS=Mus musculus GN=Rpl21 PE=1 SV=3	3.038571	0.03846	1.77048
166	sp|O88967|YMEL1_MOUSE	ATP-dependent zinc metalloprotease YME1L1 OS=Mus musculus GN=Yme1l1 PE=1 SV	11.10675	0.00037	1.788414
167	tr|Q5NCJ9|Q5NCJ9_MOUSE	Cytochrome b-c1 complex subunit 9 OS=Mus musculus GN=Uqcr10 PE=1 SV=1	3.192441	0.03314	1.788949
168	sp|Q8BTR5|DUS28_MOUSE	Dual specificity phosphatase 28 OS=Mus musculus GN=Dusp28 PE=1 SV=1	8.297839	0.00115	1.845195
169	sp|Q9DCS9|NDUBA_MOUSE	NADH dehydrogenase [ubiquinone] 1 beta subcomplex subunit 10 OS=Mus musculu	5.727765	0.0046	1.858482
170	sp|Q8JZY4|MRRP3_MOUSE	Mitochondrial ribonuclease P protein 3 OS=Mus musculus GN=Kiaa0391 PE=2 SV=	8.740975	0.00094	1.858694
171	sp|Q9CQM5|TXD17_MOUSE	Thioredoxin domain-containing protein 17 OS=Mus musculus GN=Txndc17 PE=1 SV	3.050139	0.03802	1.861633
172	tr|Q548M4|Q548M4_MOUSE	Elongation of very long chain fatty acids protein OS=Mus musculus GN=Elovl1	3.528446	0.02426	1.873959
173	tr|Q3TST4|Q3TST4_MOUSE	Protein-serine/threonine kinase OS=Mus musculus GN=Grk5 PE=1 SV=1	3.26723	0.03087	1.879433
174	sp|O88188|LY86_MOUSE	Lymphocyte antigen 86 OS=Mus musculus GN=Ly86 PE=1 SV=1	5.548319	0.00516	1.881864
175	sp|B9EKI3|TMF1_MOUSE	TATA element modulatory factor OS=Mus musculus GN=Tmf1 PE=1 SV=2	16.52074	7.86E-05	1.882977
176	sp|P50543|S10AB_MOUSE	Protein S100-A11 OS=Mus musculus GN=S100a11 PE=1 SV=1	19.26458	4.28E-05	1.889851
177	tr|E9QNL4|E9QNL4_MOUSE	Inhibitor of nuclear factor kappa-B kinase subunit alpha OS=Mus musculus GN	6.606648	0.00272	1.908316
178	tr|F6VZG1|F6VZG1_MOUSE	Protein Gm4889 OS=Mus musculus GN=Gm4889 PE=1 SV=1	6.955217	0.00225	1.909439
179	tr|Q3TXH3|Q3TXH3_MOUSE	Lysyl oxidase OS=Mus musculus GN=Lox PE=1 SV=1	4.263347	0.01302	1.950509

180	tr|Q91YU7|Q91YU7_MOUSE	Transforming growth factor beta-3 OS=Mus musculus GN=Tgfb3 PE=1 SV=1	15.25717	0.00011	1.957005
181	sp|P11031|TCP4_MOUSE	Activated RNA polymerase II transcriptional coactivator p15 OS=Mus musculus	17.19103	6.72E-05	1.98261
182	tr|E9QND8|E9QND8_MOUSE	Atlastin-2 OS=Mus musculus GN=Atl2 PE=1 SV=1	4.871157	0.00821	2.010038
183	sp|Q9EPS3|GLCE_MOUSE	D-glucuronyl C5-epimerase OS=Mus musculus GN=Glce PE=1 SV=2	3.784666	0.01936	2.02024
184	sp|Q60760|GRB10_MOUSE	Growth factor receptor-bound protein 10 OS=Mus musculus GN=Grb10 PE=1 SV=2	9.713867	0.00063	2.046382
185	tr|Q543X6|Q543X6_MOUSE	Dual-specificity mitogen-activated protein kinase kinase 4 OS=Mus musculus (	3.26419	0.03096	2.063496
186	tr|A0A0R4J0S3|A0A0R4J0S3_MOU	Reticulon 4 interacting protein 1 OS=Mus musculus GN=Rtn4ip1 PE=1 SV=1	5.201279	0.00651	2.068604
187	sp|P30115|GSTA3_MOUSE	Glutathione S-transferase A3 OS=Mus musculus GN=Gsta3 PE=1 SV=2	5.78653	0.00443	2.070059
188	sp|Q9Z175|LOXL3_MOUSE	Lysyl oxidase homolog 3 OS=Mus musculus GN=Loxl3 PE=2 SV=2	2.905336	0.04388	2.099347
189	sp|Q8C547|HTR5B_MOUSE	HEAT repeat-containing protein 5B OS=Mus musculus GN=Heatr5b PE=1 SV=3	2.801365	0.04874	2.132626
190	sp|Q9R1X5|MRP5_MOUSE	Multidrug resistance-associated protein 5 OS=Mus musculus GN=Abcc5 PE=1 SV=:	2.803305	0.04865	2.148879
191	sp|Q08288|LYAR_MOUSE	Cell growth-regulating nucleolar protein OS=Mus musculus GN=Lyar PE=1 SV=2	2.877601	0.04512	2.190228
192	tr|G5E899|G5E899_MOUSE	Plasminogen activator inhibitor 1 OS=Mus musculus GN=Serpine1 PE=1 SV=1	8.240296	0.00118	2.24124
193	tr|Q9JHF5|Q9JHF5_MOUSE	V-type proton ATPase subunit a OS=Mus musculus GN=Tcirg1 PE=1 SV=1	3.416222	0.02687	2.265528
194	tr|A2ADR8|A2ADR8_MOUSE	Nuclear inhibitor of protein phosphatase 1 OS=Mus musculus GN=Ppp1r8 PE=1 S'	4.574416	0.01023	2.34687
195	tr|A0A140LHQ8|A0A140LHQ8_MOU	Phosphatidylinositol-binding clathrin assembly protein (Fragment) OS=Mus mu:	16.2067	8.48E-05	2.355866
196	tr|Q3TXT8|Q3TXT8_MOUSE	Ninjurin 1 OS=Mus musculus GN=Ninj1 PE=1 SV=1	2.93515	0.04259	2.363949
197	sp|Q921L5|COG2_MOUSE	Conserved oligomeric Golgi complex subunit 2 OS=Mus musculus GN=Cog2 PE=1 S'	3.838559	0.01848	2.377134
198	sp|Q9DBW3|NATD1_MOUSE	Protein NATD1 OS=Mus musculus GN=Natd1 PE=1 SV=1	3.299512	0.02995	2.524545
199	sp|Q3U5F4|YRDC_MOUSE	YrdC domain-containing protein, mitochondrial OS=Mus musculus GN=Yrdc PE=1 :	4.134326	0.01444	2.579606
200	sp|Q9Z0G0|GIPC1_MOUSE	PDZ domain-containing protein GIPC1 OS=Mus musculus GN=Gipc1 PE=1 SV=1	16.18205	8.53E-05	2.660146
201	sp|Q91WF7|FIG4_MOUSE	Polyphosphoinositide phosphatase OS=Mus musculus GN=Fig4 PE=1 SV=1	3.026411	0.03892	2.726475
202	sp|Q8R2G6|CCD80_MOUSE	Coiled-coil domain-containing protein 80 OS=Mus musculus GN=Ccdc80 PE=1 SV=:	3.548737	0.02383	2.781258
203	sp|Q6NSR8|PEPL1_MOUSE	Probable aminopeptidase NPEPL1 OS=Mus musculus GN=Npepl1 PE=1 SV=1	8.392181	0.0011	2.860682
204	sp|O35929|REM1_MOUSE	GTP-binding protein REM 1 OS=Mus musculus GN=Rem1 PE=1 SV=1	3.16906	0.03389	2.876605
205	tr|Q542G9|Q542G9_MOUSE	Annexin OS=Mus musculus GN=Anxa2 PE=1 SV=1	14.50147	0.00013	2.924354
206	tr|Q5F227|Q5F227_MOUSE	Proton-coupled amino acid transporter 1 OS=Mus musculus GN=Slc36a1 PE=1 SV=	2.979632	0.04075	2.930395
207	tr|Q58E35|Q58E35_MOUSE	60S acidic ribosomal protein P1 OS=Mus musculus GN=Rplp1 PE=1 SV=1	4.798518	0.00866	3.06044
208	tr|E9PX68|E9PX68_MOUSE	Protein Slc4a1ap OS=Mus musculus GN=Slc4a1ap PE=1 SV=1	5.382853	0.00576	3.159126
209	sp|Q8BSP2|CNDH2_MOUSE	Condensin-2 complex subunit H2 OS=Mus musculus GN=Ncaph2 PE=1 SV=1	8.273528	0.00116	3.415484
210	sp|P49935|CATH_MOUSE	Pro-cathepsin H OS=Mus musculus GN=Ctsh PE=1 SV=2	3.026563	0.03891	3.42798
211	tr|G5E8V8|G5E8V8_MOUSE	Phosphorylated adapter RNA export protein OS=Mus musculus GN=Phax PE=1 SV=1	4.393936	0.01175	3.454385
212	tr|Q545T0|Q545T0_MOUSE	Cathepsin K OS=Mus musculus GN=Ctsk PE=1 SV=1	5.878336	0.00418	3.566021
213	sp|Q9D7J6|DNSL1_MOUSE	Deoxyribonuclease-1-like 1 OS=Mus musculus GN=Dnase1l1 PE=2 SV=3	3.443249	0.02622	3.655093
214	sp|Q9DCS2|CP013_MOUSE	UPF0585 protein C16orf13 homolog OS=Mus musculus PE=1 SV=1	3.951502	0.0168	3.786455
215	sp|Q61711|SIAL_MOUSE	Bone sialoprotein 2 OS=Mus musculus GN=Ibsp PE=2 SV=2	5.591688	0.00502	3.924735
216	sp|Q8CFG0|SULF2_MOUSE	Extracellular sulfatase Sulf-2 OS=Mus musculus GN=Sulf2 PE=2 SV=2	5.797792	0.0044	3.951961
217	tr|A2AFF6|A2AFF6_MOUSE	Cohesin subunit SA-2 OS=Mus musculus GN=Stag2 PE=1 SV=1	6.15095	0.00354	4.035
218	sp|Q9WU81|G6PT3_MOUSE	Glucose-6-phosphate exchanger SLC37A2 OS=Mus musculus GN=Slc37a2 PE=1 SV=1	8.356229	0.00112	4.115811
219	tr|A0A0R4J0T0|A0A0R4J0T0_MOU	Iron-sulfur cluster co-chaperone protein HscB, mitochondrial OS=Mus musculu:	13.50925	0.00017	4.39077
220	tr|Q3UP36|Q3UP36_MOUSE	B-cell receptor CD22 OS=Mus musculus GN=Cd22 PE=1 SV=1	5.661268	0.0048	4.750679
221	tr|G3UZ30|G3UZ30_MOUSE	Protein phosphatase 1 regulatory subunit 11 (Fragment) OS=Mus musculus GN=P	4.876466	0.00818	5.062154
222	sp|Q6RI63|F120B_MOUSE	Constitutive coactivator of peroxisome proliferator-activated receptor gamm:	17.42043	6.37E-05	5.619428
223	tr|A2AQR0|A2AQR0_MOUSE	Glycerol-3-phosphate dehydrogenase OS=Mus musculus GN=Gpd2 PE=1 SV=1	12.40653	0.00024	5.739588
224	tr|Q3UH28|Q3UH28_MOUSE	Protein Zmynd8 OS=Mus musculus GN=Zmynd8 PE=1 SV=1	11.03696	0.00038	5.963707
225	tr|Q4KMS1|Q4KMS1_MOUSE	Tripartite motif-containing 44 OS=Mus musculus GN=Trim44 PE=1 SV=1	3.038503	0.03846	6.446098
226	tr|D3Z132|D3Z132_MOUSE	THO complex subunit 6 homolog OS=Mus musculus GN=Thoc6 PE=1 SV=1	12.00031	0.00028	6.610176
227	sp|Q9DBL7|COASY_MOUSE	Bifunctional coenzyme A synthase OS=Mus musculus GN=Coasy PE=1 SV=2	7.543514	0.00165	6.662744
228	tr|A0A0R4J1E8|A0A0R4J1E8_MOU	Putative deoxyribonuclease TATDN1 OS=Mus musculus GN=Tatdn1 PE=1 SV=1	13.71353	0.00016	7.14407
229	sp|Q8BR90|CE051_MOUSE	UPF0600 protein C5orf51 homolog OS=Mus musculus PE=1 SV=1	3.282404	0.03043	9.64142
230	sp|Q6ZPS6|AKIB1_MOUSE	Ankyrin repeat and IBR domain-containing protein 1 OS=Mus musculus GN=Ankib	16.27739	8.34E-05	10.68508
231	sp|Q99KU0|VMP1_MOUSE	Vacuole membrane protein 1 OS=Mus musculus GN=Vmp1 PE=1 SV=2	5.617646	0.00494	14.2222
232	tr|A2AQJ8|A2AQJ8_MOUSE	Neutral alpha-glucosidase C OS=Mus musculus GN=Ganc PE=1 SV=1	31.75199	5.86E-06	52.82677

Row	Peak Name	Group	t-value	p-value	Fold Change
1	sp|P62315|SMD1_MOUSE	Small nuclear ribonucleoprotein Sm D1 OS=Mus musculus GN=Snrpd1 PE=1	-97.3626	6.67E-08	0.077751
2	sp|P39688|FYN_MOUSE	Tyrosine-protein kinase Fyn OS=Mus musculus GN=Fyn PE=1 SV=4	-17.7321	5.94E-05	0.108259
3	tr|Q8CFT3|Q8CFT3_MOUSE	Nerve growth factor receptor (TNFR superfamily, member 16) OS=Mus mus	-17.6046	6.11E-05	0.178581
4	sp|Q61711|SIAL_MOUSE	Bone sialoprotein 2 OS=Mus musculus GN=Ibsp PE=2 SV=2	-3.4583	0.02586	0.187501
5	tr|E9QNG6|E9QNG6_MOUSE	Sorting nexin-13 OS=Mus musculus GN=Snx13 PE=1 SV=2	-7.48177	0.00171	0.196015
6	tr|Q3UP36|Q3UP36_MOUSE	B-cell receptor CD22 OS=Mus musculus GN=Cd22 PE=1 SV=1	-6.08426	0.00369	0.20523
7	sp|Q6PB93|GALT2_MOUSE	Polypeptide N-acetylgalactosaminyltransferase 2 OS=Mus musculus GN=Ga	-5.79546	0.00441	0.210522
8	sp|Q9CY57|CHTOP_MOUSE	Chromatin target of PRMT1 protein OS=Mus musculus GN=Chtop PE=1 SV=2	-3.94375	0.01691	0.228199
9	sp|Q923W1|TGS1_MOUSE	Trimethylguanosine synthase OS=Mus musculus GN=Tgs1 PE=1 SV=2	-3.19646	0.03301	0.248182
10	sp|Q8K2C6|SIR5_MOUSE	NAD-dependent protein deacylase sirtuin-5, mitochondrial OS=Mus muscu	-7.68682	0.00154	0.251441
11	tr|E9QP49|E9QP49_MOUSE	EH domain-binding protein 1-like protein 1 OS=Mus musculus GN=Ehbp1l1	-6.12054	0.00361	0.281315
12	sp|Q8C547|HTR5B_MOUSE	HEAT repeat-containing protein 5B OS=Mus musculus GN=Heatr5b PE=1 SV=	-9.80841	0.00061	0.28147
13	sp|Q9WU81|G6PT3_MOUSE	Glucose-6-phosphate exchanger SLC37A2 OS=Mus musculus GN=Slc37a2 PE=1	-11.525	0.00032	0.318731
14	sp|O08692|NGP_MOUSE	Neutrophilic granule protein OS=Mus musculus GN=Ngp PE=1 SV=1	-7.6164	0.0016	0.321321
15	sp|Q61176|ARGI1_MOUSE	Arginase-1 OS=Mus musculus GN=Arg1 PE=1 SV=1	-18.312	5.23E-05	0.323899
16	sp|Q8BH61|F13A_MOUSE	Coagulation factor XIII A chain OS=Mus musculus GN=F13a1 PE=1 SV=3	-14.2454	0.00014	0.33825
17	sp|Q91VC9|GHITM_MOUSE	Growth hormone-inducible transmembrane protein OS=Mus musculus GN=Ghi	-2.87482	0.04525	0.360066
18	sp|Q3U5F4|YRDC_MOUSE	YrdC domain-containing protein, mitochondrial OS=Mus musculus GN=Yrdc	-3.13828	0.03491	0.39391
19	sp|O88967|YMEL1_MOUSE	ATP-dependent zinc metalloprotease YME1L1 OS=Mus musculus GN=Yme1l1 P	-6.75335	0.00251	0.397618
20	sp|Q99LJ7|RCBT2_MOUSE	RCC1 and BTB domain-containing protein 2 OS=Mus musculus GN=Rcbtb2 PE	-2.84217	0.04676	0.399648
21	sp|P54103|DNJC2_MOUSE	DnaJ homolog subfamily C member 2 OS=Mus musculus GN=Dnajc2 PE=1 SV=2	-2.94672	0.04211	0.401301
22	sp|Q8CH25|SLTM_MOUSE	SAFB-like transcription modulator OS=Mus musculus GN=Sltm PE=1 SV=1	-3.59247	0.02291	0.430559
23	tr|Q4FK35|Q4FK35_MOUSE	Tetraspanin OS=Mus musculus GN=Cd37 PE=1 SV=1	-4.5699	0.01026	0.432898
24	tr|A2A6J7|A2A6J7_MOUSE	Lymphocyte-specific protein 1 OS=Mus musculus GN=Lsp1 PE=1 SV=1	-12.657	0.00022	0.452915
25	tr|Q543T1|Q543T1_MOUSE	Prostaglandin G/H synthase 1 OS=Mus musculus GN=Ptgs1 PE=1 SV=1	-7.25018	0.00192	0.453276
26	sp|P47199|QOR_MOUSE	Quinone oxidoreductase OS=Mus musculus GN=Cryz PE=1 SV=1	-2.99142	0.04028	0.453601
27	tr|E9PUW7|E9PUW7_MOUSE	Exportin-7 OS=Mus musculus GN=Xpo7 PE=1 SV=1	-6.9449	0.00226	0.469355
28	tr|Q0PD50|Q0PD50_MOUSE	RAB8A, member RAS oncogene family, isoform CRA_a OS=Mus musculus GN=R	-2.78605	0.04951	0.472458
29	sp|Q9R0P9|UCHL1_MOUSE	Ubiquitin carboxyl-terminal hydrolase isozyme L1 OS=Mus musculus GN=U	-5.47974	0.0054	0.475547
30	tr|Q5SV64|Q5SV64_MOUSE	Myosin-10 OS=Mus musculus GN=Myh10 PE=1 SV=1	-7.56193	0.00164	0.48086
31	sp|Q3UHQ6|DOP2_MOUSE	Protein dopey-2 OS=Mus musculus GN=Dopey2 PE=1 SV=3	-2.95007	0.04197	0.490435
32	tr|A0A0R4J1K9|A0A0R4J1K9_MOU	Anion exchange protein OS=Mus musculus GN=Slc4a2 PE=1 SV=1	-2.99459	0.04016	0.493948
33	sp|P05201|AATC_MOUSE	Aspartate aminotransferase, cytoplasmic OS=Mus musculus GN=Got1 PE=1	-7.57118	0.00163	0.496591
34	sp|Q8BZ36|RINT1_MOUSE	RAD50-interacting protein 1 OS=Mus musculus GN=Rint1 PE=1 SV=2	-13.1921	0.00019	0.503764
35	sp|Q91W39|NCOA5_MOUSE	Nuclear receptor coactivator 5 OS=Mus musculus GN=Ncoa5 PE=1 SV=1	-3.09992	0.03622	0.513838
36	tr|Q52L78|Q52L78_MOUSE	Alpha-crystallin B chain OS=Mus musculus GN=Cryab PE=1 SV=1	-8.57856	0.00101	0.516178
37	tr|E9QND8|E9QND8_MOUSE	Atlastin-2 OS=Mus musculus GN=Atl2 PE=1 SV=1	-4.32131	0.01243	0.523659
38	tr|E9QLW5|E9QLW5_MOUSE	Insulin-like growth factor II OS=Mus musculus GN=Igf2 PE=1 SV=1	-13.7681	0.00016	0.528793
39	tr|E9QNL8|E9QNL8_MOUSE	Integrin alpha-L OS=Mus musculus GN=Itgal PE=1 SV=1	-5.7055	0.00467	0.528822
40	tr|G3X934|G3X934_MOUSE	MCG115964 OS=Mus musculus GN=Wdr70 PE=1 SV=1	-3.32807	0.02916	0.533736
41	tr|B2RUC7|B2RUC7_MOUSE	Serine-threonine kinase receptor-associated protein OS=Mus musculus G	-6.45976	0.00296	0.541297
42	sp|Q91XE8|TM205_MOUSE	Transmembrane protein 205 OS=Mus musculus GN=Tmem205 PE=1 SV=1	-3.25378	0.03126	0.555413
43	sp|Q80X85|RT07_MOUSE	28S ribosomal protein S7, mitochondrial OS=Mus musculus GN=Mrps7 PE=1	-3.78544	0.01934	0.556642
44	tr|Q3U3V1|Q3U3V1_MOUSE	Coagulation factor X OS=Mus musculus GN=F10 PE=1 SV=1	-6.14768	0.00355	0.557475
45	tr|Q547B5|Q547B5_MOUSE	Osteopontin OS=Mus musculus GN=Spp1 PE=1 SV=1	-9.81896	0.0006	0.557779
46	tr|Q0PD34|Q0PD34_MOUSE	RAB22A, member RAS oncogene family, isoform CRA_a OS=Mus musculus GN=	-3.92447	0.01718	0.562435
47	sp|O88783|FA5_MOUSE	Coagulation factor V OS=Mus musculus GN=F5 PE=1 SV=1	-11.4246	0.00033	0.565967
48	sp|O54824|IL16_MOUSE	Pro-interleukin-16 OS=Mus musculus GN=Il16 PE=1 SV=3	-8.87911	0.00089	0.571642
49	tr|Q3TV21|Q3TV21_MOUSE	Frataxin OS=Mus musculus GN=Fxn PE=1 SV=1	-2.98898	0.04038	0.574399
50	tr|Q3TDI2|Q3TDI2_MOUSE	Wolfram syndrome 1 homolog (Human) OS=Mus musculus GN=Wfs1 PE=1 SV=1	-5.78981	0.00442	0.579813
51	tr|Q3U6G0|Q3U6G0_MOUSE	Cytochrome b-245 heavy chain OS=Mus musculus GN=Cybb PE=1 SV=1	-3.72434	0.0204	0.58243
52	sp|P51880|FABP7_MOUSE	Fatty acid-binding protein, brain OS=Mus musculus GN=Fabp7 PE=1 SV=2	-12.5245	0.00023	0.584764
53	tr|Q6NSP9|Q6NSP9_MOUSE	High mobility group protein HMGI-C OS=Mus musculus GN=Hmga2 PE=1 SV=1	-2.92967	0.04283	0.589216
54	sp|Q920A7|AFG31_MOUSE	AFG3-like protein 1 OS=Mus musculus GN=Afg3l1 PE=1 SV=2	-6.1346	0.00358	0.589799
55	tr|G5E899|G5E899_MOUSE	Plasminogen activator inhibitor 1 OS=Mus musculus GN=Serpine1 PE=1 SV	-7.02434	0.00216	0.592664
56	sp|Q05769|PGH2_MOUSE	Prostaglandin G/H synthase 2 OS=Mus musculus GN=Ptgs2 PE=1 SV=1	-4.96409	0.00768	0.593097
57	sp|Q8K2J7|RELL1_MOUSE	RELT-like protein 1 OS=Mus musculus GN=Rell1 PE=2 SV=2	-2.94075	0.04236	0.595572
58	sp|B9EKI3|TMF1_MOUSE	TATA element modulatory factor OS=Mus musculus GN=Tmf1 PE=1 SV=2	-12.3319	0.00025	0.596627
59	sp|P01942|HBA_MOUSE	Hemoglobin subunit alpha OS=Mus musculus GN=Hba PE=1 SV=2	-9.26727	0.00075	0.608148
60	sp|Q149L6|DJB14_MOUSE	DnaJ homolog subfamily B member 14 OS=Mus musculus GN=Dnajb14 PE=2 SV	-6.07536	0.00371	0.611081
61	sp|Q91VM9|IPYR2_MOUSE	Inorganic pyrophosphatase 2, mitochondrial OS=Mus musculus GN=Ppa2 PE	-4.48762	0.01093	0.613376
62	sp|Q99NH8|TREM2_MOUSE	Triggering receptor expressed on myeloid cells 2 OS=Mus musculus GN=T	-3.30963	0.02966	0.616903
63	sp|Q8K4L3|SVIL_MOUSE	Supervillin OS=Mus musculus GN=Svil PE=1 SV=1	-3.38492	0.02766	0.617813
64	tr|Q542U3|Q542U3_MOUSE	General transcription factor IIH subunit 4 OS=Mus musculus GN=Gtf2h4	-5.85987	0.00423	0.618927
65	sp|Q9Z204|HNRPC_MOUSE	Heterogeneous nuclear ribonucleoproteins C1/C2 OS=Mus musculus GN=Hnr	-3.9536	0.01677	0.619769
66	sp|Q8K449|ABCA9_MOUSE	ATP-binding cassette sub-family A member 9 OS=Mus musculus GN=Abca9 P	-7.98351	0.00133	0.623331
67	tr|Q5FWJ3|Q5FWJ3_MOUSE	Vimentin OS=Mus musculus GN=Vim PE=1 SV=1	-9.9675	0.00057	0.625867
68	sp|Q91VW5|GOGA4_MOUSE	Golgin subfamily A member 4 OS=Mus musculus GN=Golga4 PE=1 SV=2	-3.04029	0.03839	0.628531
69	sp|Q9R118|HTRA1_MOUSE	Serine protease HTRA1 OS=Mus musculus GN=Htra1 PE=1 SV=2	-13.4987	0.00017	0.630268
70	tr|B2RX66|B2RX66_MOUSE	MCG124812 OS=Mus musculus GN=Taok1 PE=1 SV=1	-3.23194	0.03192	0.631448
71	tr|A0A0R4J0S3|A0A0R4J0S3_MOU	Reticulon 4 interacting protein 1 OS=Mus musculus GN=Rtn4ip1 PE=1 SV=	-3.07479	0.03712	0.632465
72	tr|Q544K9|Q544K9_MOUSE	Uridine 5'-monophosphate synthase OS=Mus musculus GN=Umps PE=1 SV=1	-3.33476	0.02898	0.632697
73	sp|Q8BPU7|ELMO1_MOUSE	Engulfment and cell motility protein 1 OS=Mus musculus GN=Elmo1 PE=1	-3.59774	0.0228	0.63352
74	tr|Q542G9|Q542G9_MOUSE	Annexin OS=Mus musculus GN=Anxa2 PE=1 SV=1	-4.22293	0.01345	0.639151
75	sp|Q8BX80|ENASE_MOUSE	Cytosolic endo-beta-N-acetylglucosaminidase OS=Mus musculus GN=Engase	-6.48802	0.00291	0.648343
76	sp|Q03145|EPHA2_MOUSE	Ephrin type-A receptor 2 OS=Mus musculus GN=Epha2 PE=1 SV=3	-5.21357	0.00646	0.648512
77	tr|Q3UG85|Q3UG85_MOUSE	Progressive ankylosis OS=Mus musculus GN=Ank PE=1 SV=1	-6.40748	0.00305	0.652012
78	tr|E9Q456|E9Q456_MOUSE	Tropomyosin alpha-1 chain OS=Mus musculus GN=Tpm1 PE=1 SV=1	-10.4157	0.00048	0.654681
79	sp|Q9DB15|RM12_MOUSE	39S ribosomal protein L12, mitochondrial OS=Mus musculus GN=Mrpl12 PE	-3.08651	0.0367	0.657478
80	sp|Q8CGC7|SYEP_MOUSE	Bifunctional glutamate/proline--tRNA ligase OS=Mus musculus GN=Eprs P	-3.13829	0.03491	0.659141
81	sp|Q3UQN2|FCHO2_MOUSE	F-BAR domain only protein 2 OS=Mus musculus GN=Fcho2 PE=1 SV=1	-3.74074	0.02011	0.660203
82	tr|B1AU74|B1AU74_MOUSE	Motile sperm domain-containing protein 2 OS=Mus musculus GN=Mospd2 PE	-13.0342	0.0002	0.660294
83	tr|Q8BW87|Q8BW87_MOUSE	AP-1 complex subunit sigma-2 OS=Mus musculus GN=Ap1s2 PE=1 SV=1	-3.33916	0.02886	0.66036
84	sp|P18872|GNAO_MOUSE	Guanine nucleotide-binding protein G(o) subunit alpha OS=Mus musculus	2.923001	0.04311	1.500536
85	sp|Q91Z96|BMP2K_MOUSE	BMP-2-inducible protein kinase OS=Mus musculus GN=Bmp2k PE=1 SV=1	5.950127	0.004	1.505977
86	sp|O55239|NNMT_MOUSE	Nicotinamide N-methyltransferase OS=Mus musculus GN=Nnmt PE=1 SV=1	5.305402	0.00606	1.506064
87	tr|Q546G4|Q546G4_MOUSE	Albumin 1 OS=Mus musculus GN=Alb PE=1 SV=1	23.42539	1.97E-05	1.509613

88	tr|A0A0R4J139|A0A0R4J139_MOU	Methionine sulfoxide reductase B3 OS=Mus musculus GN=Msrb3 PE=1 SV=1	7.21365	0.00196	1.513661
89	sp|Q8BXL7|ARFRP_MOUSE	ADP-ribosylation factor-related protein 1 OS=Mus musculus GN=Arfrp1 P	4.863458	0.00826	1.515143
90	tr|G3X977|G3X977_MOUSE	Inter-alpha trypsin inhibitor, heavy chain 2 OS=Mus musculus GN=Itih2	4.866432	0.00824	1.517144
91	sp|P24472|GSTA4_MOUSE	Glutathione S-transferase A4 OS=Mus musculus GN=Gsta4 PE=1 SV=3	13.92311	0.00015	1.51773
92	tr|F8WGM3|F8WGM3_MOUSE	Epsilon-sarcoglycan OS=Mus musculus GN=Sgce PE=1 SV=2	4.371595	0.01195	1.520856
93	tr|H3BL37|H3BL37_MOUSE	Treacle protein OS=Mus musculus GN=Tcof1 PE=1 SV=1	5.494525	0.00535	1.52529
94	sp|Q8VHJ5|MARK1_MOUSE	Serine/threonine-protein kinase MARK1 OS=Mus musculus GN=Mark1 PE=1 S	4.239036	0.01327	1.530366
95	tr|Q9D8S5|Q9D8S5_MOUSE	MCG7614, isoform CRA_c OS=Mus musculus GN=Srsf5 PE=1 SV=1	7.588033	0.00162	1.530729
96	sp|Q64133|AOFA_MOUSE	Amine oxidase [flavin-containing] A OS=Mus musculus GN=Maoa PE=1 SV=3	8.103964	0.00126	1.533291
97	sp|Q03350|TSP2_MOUSE	Thrombospondin-2 OS=Mus musculus GN=Thbs2 PE=1 SV=2	4.447199	0.01127	1.54113
98	sp|O88492|PLIN4_MOUSE	Perilipin-4 OS=Mus musculus GN=Plin4 PE=1 SV=2	3.782708	0.01939	1.541356
99	tr|A0A0R4J027|A0A0R4J027_MOU	Protein Acod1 OS=Mus musculus GN=Acod1 PE=1 SV=1	7.556716	0.00164	1.549347
100	tr|Q8C6B0|Q8C6B0_MOUSE	MCG20149, isoform CRA_a OS=Mus musculus GN=Mettl7a1 PE=1 SV=1	10.47644	0.00047	1.559379
101	sp|P19324|SERPH_MOUSE	Serpin H1 OS=Mus musculus GN=Serpinh1 PE=1 SV=3	13.79202	0.00016	1.560214
102	tr|B8JJN0|B8JJN0_MOUSE	Protein Gm20547 OS=Mus musculus GN=Gm20547 PE=3 SV=1	6.832999	0.0024	1.569979
103	sp|Q8K2B0|SC65_MOUSE	Synaptonemal complex protein SC65 OS=Mus musculus GN=P3h4 PE=2 SV=1	3.351302	0.02853	1.579896
104	sp|Q8C1E7|T120A_MOUSE	Transmembrane protein 120A OS=Mus musculus GN=Tmem120a PE=1 SV=1	2.859369	0.04596	1.584256
105	sp|Q9WTQ5|AKA12_MOUSE	A-kinase anchor protein 12 OS=Mus musculus GN=Akap12 PE=1 SV=1	26.40362	1.22E-05	1.587978
106	tr|E9Q804|E9Q804_MOUSE	Ankyrin repeat domain-containing protein 17 OS=Mus musculus GN=Ankrd1	5.838785	0.00429	1.597843
107	sp|P97452|BOP1_MOUSE	Ribosome biogenesis protein BOP1 OS=Mus musculus GN=Bop1 PE=1 SV=1	5.786308	0.00443	1.605938
108	sp|Q9Z1T1|AP3B1_MOUSE	AP-3 complex subunit beta-1 OS=Mus musculus GN=Ap3b1 PE=1 SV=2	11.21715	0.00036	1.615086
109	sp|P09528|FRIH_MOUSE	Ferritin heavy chain OS=Mus musculus GN=Fth1 PE=1 SV=2	11.22417	0.00036	1.615218
110	sp|Q569Z5|DDX46_MOUSE	Probable ATP-dependent RNA helicase DDX46 OS=Mus musculus GN=Ddx46 PE	11.60098	0.00032	1.61642
111	sp|Q62383|SPT6H_MOUSE	Transcription elongation factor SPT6 OS=Mus musculus GN=Supt6h PE=1 S	3.881814	0.01781	1.622373
112	tr|Q9DCY1|Q9DCY1_MOUSE	Peptidyl-prolyl cis-trans isomerase OS=Mus musculus GN=Ppib PE=1 SV=1	2.909656	0.04369	1.627048
113	sp|Q91XE4|ACY3_MOUSE	N-acyl-aromatic-L-amino acid amidohydrolase (carboxylate-forming) OS=	4.334435	0.01231	1.629783
114	sp|Q9CQW9|IFM3_MOUSE	Interferon-induced transmembrane protein 3 OS=Mus musculus GN=Ifitm3	5.330851	0.00596	1.636629
115	sp|Q9QZS3|NUMB_MOUSE	Protein numb homolog OS=Mus musculus GN=Numb PE=1 SV=1	3.819177	0.01879	1.646144
116	sp|Q80YS6|AFAP1_MOUSE	Actin filament-associated protein 1 OS=Mus musculus GN=Afap1 PE=1 SV=	8.772531	0.00093	1.647015
117	sp|Q80SW1|SAHH2_MOUSE	Putative adenosylhomocysteinase 2 OS=Mus musculus GN=Ahcyl1 PE=1 SV=1	4.394688	0.01174	1.649432
118	tr|F8WI64|F8WI64_MOUSE	Vacuolar protein sorting-associated protein 8 homolog OS=Mus musculus	5.201591	0.00651	1.65659
119	tr|F8VPL2|F8VPL2_MOUSE	Phosphatidylinositol 4-phosphate 3-kinase C2 domain-containing subuni	17.2413	6.64E-05	1.661377
120	sp|Q04857|CO6A1_MOUSE	Collagen alpha-1(VI) chain OS=Mus musculus GN=Col6a1 PE=1 SV=1	5.188899	0.00657	1.67948
121	sp|Q3TWW8|SRSF6_MOUSE	Serine/arginine-rich splicing factor 6 OS=Mus musculus GN=Srsf6 PE=1	21.74435	2.65E-05	1.682904
122	sp|Q9DBF1|AL7A1_MOUSE	Alpha-aminoadipic semialdehyde dehydrogenase OS=Mus musculus GN=Aldh7	9.861229	0.00059	1.683848
123	sp|O88428|PAPS2_MOUSE	Bifunctional 3'-phosphoadenosine 5'-phosphosulfate synthase 2 OS=Mus	5.767184	0.00449	1.691024
124	tr|Q3TWI2|Q3TWI2_MOUSE	Sarcosine dehydrogenase OS=Mus musculus GN=Sardh PE=1 SV=1	3.159747	0.03419	1.700483
125	sp|D3Z4S3|PTRD1_MOUSE	Putative peptidyl-tRNA hydrolase PTRHD1 OS=Mus musculus GN=Ptrhd1 PE=	9.365627	0.00072	1.701274
126	sp|Q9QXY6|EHD3_MOUSE	EH domain-containing protein 3 OS=Mus musculus GN=Ehd3 PE=1 SV=2	4.188342	0.01382	1.713827
127	sp|Q9D4H2|GCC1_MOUSE	GRIP and coiled-coil domain-containing protein 1 OS=Mus musculus GN=G	4.419913	0.01151	1.718156
128	sp|Q9D6X6|PRS23_MOUSE	Serine protease 23 OS=Mus musculus GN=Prss23 PE=2 SV=2	3.101985	0.03615	1.728974
129	sp|P41233|ABCA1_MOUSE	ATP-binding cassette sub-family A member 1 OS=Mus musculus GN=Abca1 P	4.653082	0.00964	1.734812
130	sp|Q62093|SRSF2_MOUSE	Serine/arginine-rich splicing factor 2 OS=Mus musculus GN=Srsf2 PE=1	14.82323	0.00012	1.740957
131	tr|G5E8J0|G5E8J0_MOUSE	Neurogenic locus notch homolog protein 2 OS=Mus musculus GN=Notch2 PE	3.417274	0.02685	1.744498
132	sp|Q61469|PLPP1_MOUSE	Phospholipid phosphatase 1 OS=Mus musculus GN=Plpp1 PE=1 SV=1	10.93821	0.0004	1.766995
133	sp|Q99JI1|MSTN1_MOUSE	Musculoskeletal embryonic nuclear protein 1 OS=Mus musculus GN=Mustn1	4.508908	0.01075	1.778602
134	tr|A2AEX8|A2AEX8_MOUSE	Four and a half LIM domains 1, isoform CRA_b OS=Mus musculus GN=Fhl1	29.15738	8.24E-06	1.780061
135	sp|P21460|CYTC_MOUSE	Cystatin-C OS=Mus musculus GN=Cst3 PE=1 SV=2	4.735345	0.00907	1.808995
136	sp|Q05D44|IF2P_MOUSE	Eukaryotic translation initiation factor 5B OS=Mus musculus GN=Eif5b	18.28857	5.26E-05	1.813834
137	tr|A0A0R4J0S1|A0A0R4J0S1_MOU	Cdc42 effector protein 1 OS=Mus musculus GN=Cdc42ep1 PE=1 SV=1	10.86297	0.00041	1.81402
138	sp|P47879|IBP4_MOUSE	Insulin-like growth factor-binding protein 4 OS=Mus musculus GN=Igfbp	3.99876	0.01615	1.828359
139	sp|Q60847|COCA1_MOUSE	Collagen alpha-1(XII) chain OS=Mus musculus GN=Col12a1 PE=2 SV=3	12.68315	0.00022	1.833514
140	tr|H7BX95|H7BX95_MOUSE	Serine/arginine-rich-splicing factor 1 OS=Mus musculus GN=Srsf1 PE=1	23.78738	1.85E-05	1.851967
141	tr|A0A0G2JGQ6|A0A0G2JGQ6_MOU	Serine/threonine-protein kinase DCLK1 OS=Mus musculus GN=Dclk1 PE=1 S	5.371398	0.0058	1.89624
142	sp|Q08879|FBLN1_MOUSE	Fibulin-1 OS=Mus musculus GN=Fbln1 PE=1 SV=2	8.953321	0.00086	1.902039
143	tr|G3X961|G3X961_MOUSE	Kelch domain containing 4, isoform CRA_a OS=Mus musculus GN=Klhdc4 PE	12.81855	0.00021	1.937362
144	sp|Q8VDZ4|ZDHC5_MOUSE	Palmitoyltransferase ZDHHC5 OS=Mus musculus GN=Zdhhc5 PE=1 SV=1	3.333243	0.02902	2.000191
145	sp|Q9ES28|ARHG7_MOUSE	Rho guanine nucleotide exchange factor 7 OS=Mus musculus GN=Arhgef7 P	3.611137	0.02253	2.018579
146	tr|Q3TVI5|Q3TVI5_MOUSE	Collagen alpha-1(III) chain OS=Mus musculus GN=Col3a1 PE=1 SV=1	20.95909	3.06E-05	2.042948
147	tr|B2RQZ6|B2RQZ6_MOUSE	G protein-regulated inducer of neurite outgrowth 3 OS=Mus musculus GN	4.252298	0.01313	2.044175
148	sp|Q3V3R4|ITA1_MOUSE	Integrin alpha-1 OS=Mus musculus GN=Itga1 PE=1 SV=2	25.7489	1.35E-05	2.04861
149	sp|Q9Z0M5|LICH_MOUSE	Lysosomal acid lipase/cholesteryl ester hydrolase OS=Mus musculus GN=	3.061796	0.03759	2.163432
150	tr|Q3TBV5|Q3TBV5_MOUSE	Interleukin-1 receptor antagonist protein OS=Mus musculus GN=Il1rn PE	10.2027	0.00052	2.1982
151	tr|D3Z4C0|D3Z4C0_MOUSE	FERM, RhoGEF and pleckstrin domain-containing protein 2 OS=Mus muscul	4.599953	0.01003	2.210855
152	tr|A0A087WQZ9|A0A087WQZ9_MOU	Lymphocyte antigen 96 OS=Mus musculus GN=Ly96 PE=1 SV=1	3.181438	0.03349	2.261626
153	sp|Q9D281|NXP20_MOUSE	Protein Noxp20 OS=Mus musculus GN=Fam114a1 PE=1 SV=1	3.947632	0.01685	2.276781
154	sp|Q8BJH1|ZC21A_MOUSE	Zinc finger C2HC domain-containing protein 1A OS=Mus musculus GN=Zc2h	3.694253	0.02094	2.278916
155	sp|P52623|UCK1_MOUSE	Uridine-cytidine kinase 1 OS=Mus musculus GN=Uck1 PE=1 SV=2	4.682057	0.00943	2.281345
156	tr|A0A0A6YW87|A0A0A6YW87_MOU	Cornichon homolog 4 (Drosophila), isoform CRA_a OS=Mus musculus GN=Cn	9.474944	0.00069	2.289035
157	sp|Q9WVH9|FBLN5_MOUSE	Fibulin-5 OS=Mus musculus GN=Fbln5 PE=1 SV=1	4.678444	0.00946	2.298096
158	tr|B1B1A8|B1B1A8_MOUSE	Myosin light chain kinase, smooth muscle OS=Mus musculus GN=Mylk PE=1	18.4482	5.08E-05	2.30965
159	tr|V9GXP6|V9GXP6_MOUSE	MARVEL domain-containing protein 1 (Fragment) OS=Mus musculus GN=Marv	3.508443	0.02471	2.338593
160	sp|Q3UG98|NAT9_MOUSE	N-acetyltransferase 9 OS=Mus musculus GN=Nat9 PE=1 SV=2	6.291467	0.00326	2.376894
161	tr|B1AV01|B1AV01_MOUSE	E3 ubiquitin-protein ligase Midline-1 OS=Mus musculus GN=Mid1 PE=1 SV	3.863678	0.01809	2.400863
162	sp|Q8BLV3|SL9A7_MOUSE	Sodium/hydrogen exchanger 7 OS=Mus musculus GN=Slc9a7 PE=1 SV=1	3.012526	0.03945	2.436641
163	tr|I7HJQ9|I7HJQ9_MOUSE	Myotubularin-related protein 1 OS=Mus musculus GN=Mtmr1 PE=1 SV=1	3.367395	0.02811	2.458896
164	sp|Q8VDW0|DX39A_MOUSE	ATP-dependent RNA helicase DDX39A OS=Mus musculus GN=Ddx39a PE=1 SV=1	12.45411	0.00024	2.542835
165	sp|O88676|MMP23_MOUSE	Matrix metalloproteinase-23 OS=Mus musculus GN=Mmp23 PE=2 SV=1	3.556023	0.02367	2.551084
166	sp|Q3UIR3|DTX3L_MOUSE	E3 ubiquitin-protein ligase DTX3L OS=Mus musculus GN=Dtx3l PE=1 SV=1	3.068275	0.03736	2.566569
167	tr|F6Q8D3|F6Q8D3_MOUSE	Phospholipid-transporting ATPase OS=Mus musculus GN=Atp11c PE=1 SV=1	3.419112	0.0268	2.689258
168	sp|Q8K2V6|IPO11_MOUSE	Importin-11 OS=Mus musculus GN=Ipo11 PE=1 SV=1	7.38593	0.00179	2.716423
169	tr|Q3UAM9|Q3UAM9_MOUSE	Endoglin OS=Mus musculus GN=Eng PE=1 SV=1	3.899943	0.01754	2.722591
170	sp|Q9R0Y5|KAD1_MOUSE	Adenylate kinase isoenzyme 1 OS=Mus musculus GN=Ak1 PE=1 SV=1	4.33716	0.01228	2.772354
171	sp|Q9QY73|TMM59_MOUSE	Transmembrane protein 59 OS=Mus musculus GN=Tmem59 PE=1 SV=2	2.93834	0.04246	2.787759
172	sp|P04918|SAA3_MOUSE	Serum amyloid A-3 protein OS=Mus musculus GN=Saa3 PE=1 SV=1	4.83115	0.00845	2.915589
173	sp|Q8C4U3|SFRP1_MOUSE	Secreted frizzled-related protein 1 OS=Mus musculus GN=Sfrp1 PE=1 SV=	2.813053	0.04817	3.021131
174	sp|Q8CFE6|S38A2_MOUSE	Sodium-coupled neutral amino acid transporter 2 OS=Mus musculus GN=Sl	4.28581	0.01279	3.090448
175	sp|P37889|FBLN2_MOUSE	Fibulin-2 OS=Mus musculus GN=Fbln2 PE=1 SV=2	14.61134	0.00013	3.095105

176	tr|A0A0R4J263|A0A0R4J263_MOU	1-acyl-sn-glycerol-3-phosphate acyltransferase OS=Mus musculus GN=Agp	3.206035	0.03271	3.149733
177	tr|A0A0U1RPI1|A0A0U1RPI1_MOU	Phospholemman OS=Mus musculus GN=Fxyd1 PE=1 SV=1	4.508905	0.01075	3.691563
178	tr|Q8BXX3|Q8BXX3_MOUSE	GPI-anchor transamidase OS=Mus musculus GN=Pigk PE=1 SV=1	3.118323	0.03558	3.802116
179	sp|O70423|AOC3_MOUSE	Membrane primary amine oxidase OS=Mus musculus GN=Aoc3 PE=1 SV=3	25.94785	1.31E-05	3.915977
180	tr|Q3UID0|Q3UID0_MOUSE	SWI/SNF complex subunit SMARCC2 OS=Mus musculus GN=Smarcc2 PE=1 SV=1	10.44605	0.00047	4.064379
181	tr|F8WHD8|F8WHD8_MOUSE	Synaptojanin-2 OS=Mus musculus GN=Synj2 PE=1 SV=1	7.275241	0.0019	4.139247
182	sp|P68181|KAPCB_MOUSE	cAMP-dependent protein kinase catalytic subunit beta OS=Mus musculus	3.120753	0.0355	4.673185
183	sp|Q76N33|STALP_MOUSE	AMSH-like protease OS=Mus musculus GN=Stambpl1 PE=1 SV=1	6.039282	0.00379	5.095218
184	tr|E9Q3Y4|E9Q3Y4_MOUSE	Lipopolysaccharide-responsive and beige-like anchor protein OS=Mus mu	7.058273	0.00213	5.790742
185	sp|P11531|DMD_MOUSE	Dystrophin OS=Mus musculus GN=Dmd PE=1 SV=3	14.86161	0.00012	9.955924
