# Supplementary figures and images for: Mettl7a alleviated bone loss in osteoporosis mice by targeting the O-GlcNAcylation of Bsp via m6A methylation
Source: Stem Cells Transl Med. 2025 Jun 25;14(7):szaf024. doi: 10.1093/stcltm/szaf024 (PMC12188527; doi:10.1093/stcltm/szaf024)

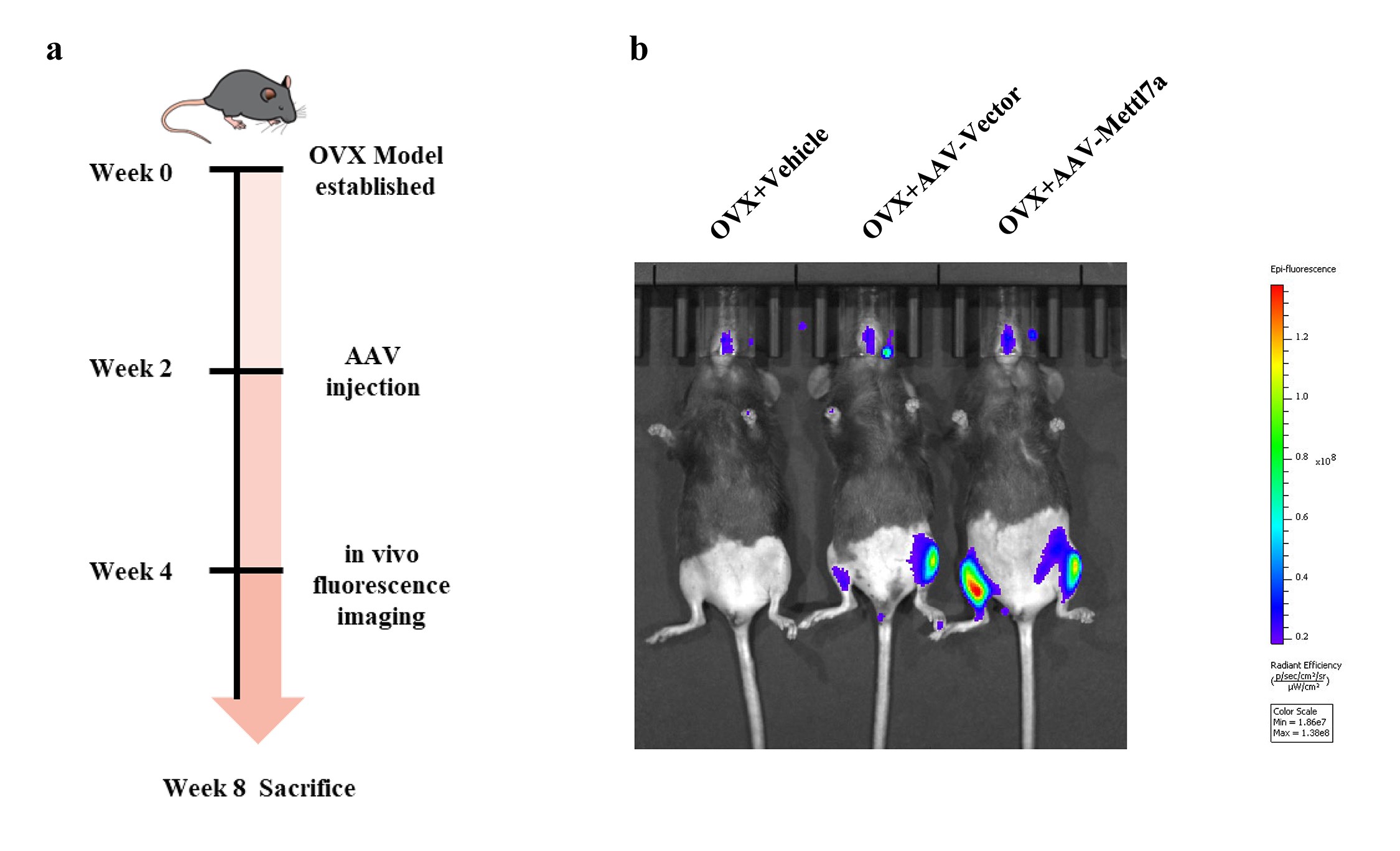

Supplement: szaf024_suppl_Supplementary_Figures_S1 [file szaf024_suppl_supplementary_figures_s1.jpeg]
